# Urban–rural disparity in global estimation of PM_2·5_ household air pollution and its attributable health burden

**DOI:** 10.1016/S2542-5196(23)00133-X

**Published:** 2023-08-07

**Authors:** Nahid Mohajeri, Shih-Che Hsu, James Milner, Jonathon Taylor, Gregor Kiesewetter, Agust Gudmundsson, Harry Kennard, Ian Hamilton, Mike Davies

**Affiliations:** a Institute of Environmental Design and Engineering, Bartlett School of Environment, Energy and Resources, University College London, London, UK; b Energy Institute, University College London, London, UK; c Department of Public Health, Environments, and Society, London School of Hygiene & Tropical Medicine, London, UK; d Department of Civil Engineering, Tampere University, Tampere, Finland; e International Institute for Applied Systems Analysis, Laxenburg, Austria; f Department of Earth Sciences, Royal Holloway, University of London, Egham, UK; g Center on Global Energy Policy, Columbia University, New York, NY, USA

## Abstract

**Background:**

Polluting fuels and inefficient stove technologies are still a leading cause of premature deaths worldwide, particularly in low-income and middle-income countries. Previous studies of global household air pollution (HAP) have neither considered the estimation of PM_2·5_ at national level nor the corresponding attributable mortality burden. Additionally, the effects of climate and ambient air pollution on the global estimation of HAP-PM_2·5_ exposure for different urban and rural settings remain largely unknown. In this study, we include climatic effects to estimate the HAP-PM_2·5_ exposure from different fuel types and stove technologies in rural and urban settings separately and the related attributable global mortality burden.

**Methods:**

Bayesian hierarchical models were developed to estimate an annual average HAP-PM_2·5_ personal exposure and HAP-PM_2·5_ indoor concentration (including both outdoor and indoor sources). Model variables were selected from sample data in 282 peer-reviewed studies drawn and updated from the WHO Global HAP dataset. The PM_2·5_ exposure coefficients from the developed model were applied to the external datasets to predict the HAP-PM_2·5_ exposure globally (personal exposure in 62 countries and indoor concentration in 69 countries). Attributable mortality rate was estimated using a comparative risk assessment approach. Using weighted averages, the national level 24 h average HAP-PM_2·5_ exposure due to polluting and clean fuels and related death rate per 100 000 population were estimated.

**Findings:**

In 2020, household use of polluting solid fuels for cooking and heating led to a national-level average personal exposure of 151 μg/m^3^ (95% CI 133–169), with rural households having an average of 171 μg/m^3^ (153–189) and urban households an average of 92 μg/m^3^ (77–106). Use of clean fuels gave rise to a national-level average personal exposure of 69 μg/m^3^ (62–76), with a rural average of 76 μg/m^3^ (69–83) and an urban average of 49 μg/m^3^ (46–53). Personal exposure-attributable premature mortality (per 100 000 population) from the use of polluting solid fuels at national level was on average 78 (95% CI 69–87), with a rural average of 82 (73–90) and an urban average of 66 (57–75). The average attributable premature mortality (per 100 000 population) from the use of clean fuels at the national level is 62 (54–70), with a rural average of 66 (58–74) and an urban average of 52 (47–57). The estimated HAP-PM_2·5_ indoor concentration shows that the use of polluting solid fuels resulted in a national-level average of 412 μg/m^3^ (95% CI 353–471), with a rural average of 514 μg/m^3^ (446–582) and an urban average of 149 μg/m^3^ (126–173). The use of clean fuels (gas and electricity) led to an average PM_2·5_ indoor concentration of 135 μg/m^3^ (117–153), with a rural average of 174 μg/m^3^ (154–195) and an urban average of 71 μg/m^3^ (63–80). Using time-weighted HAP-PM_2·5_ indoor concentrations, the attributable premature death rate (per 100 000 population) from the use of polluting solid fuels at the national level is on average 78 (95% CI 72–84), the rural average being 84 (78–91) and the urban average 60 (54–66). From the use of clean fuels, the average attributable premature death rate (per 100 000 population) at the national level is 59 (53–64), the rural average being 68 (62–74) and the urban average 45 (41–50).

**Interpretation:**

A shift from polluting to clean fuels can reduce the average PM_2·5_ personal exposure by 53% and thereby lower the death rate. For all fuel types, the estimated average HAP-PM_2·5_ personal exposure and indoor concentrations exceed the WHO's Interim Target-1 average annual threshold. Policy interventions are urgently needed to greatly increase the use of clean fuels and stove technologies by 2030 to achieve the goal of affordable clean energy access, as set by the UN in 2015, and address health inequities in urban–rural settings.

**Funding:**

Wellcome Trust, The *Lancet* Countdown, the Engineering and Physical Sciences Research Council, and the Natural Environment Research Council.

## Introduction

The estimated number of people without access to clean fuels for cooking was 2·4 billion in 2020.^1^ At the current rate of improvement, there will still be 2·1 billion people using polluting fuels and inefficient technologies in the year 2030, mostly living in low-income and middle-income countries. There are also large inequalities in access to cleaner fuels between urban and rural areas. In 2020, 14% of people in global urban areas relied on polluting fuels (eg, coal, charcoal, crop residue, animal dung, and wood), and technologies, compared with 52% of the rural population.^2^

**Research in context**

**Evidence before this study**

Existing studies recognise that the burning of polluting fuels has a substantial effect on human health worldwide but rely mostly on the proportion of the population exposed to household air pollution (HAP) from polluting fuels and inefficient technologies rather than on determining a population exposure to HAP-PM_2·5_ for households in each country. Furthermore, although existing health effect assessment studies provide country-specific estimates of the burden of disease due to HAP exposure for males and females, they do not account for the variation of HAP exposure between urban and rural settings. A recent global HAP modelling study focuses primarily on estimates of PM_2·5_ personal exposure and PM_2·5_ kitchen concentrations in urban and rural settings. That study, however, provides neither the national-level (population) HAP-PM_2·5_ exposure estimation nor the corresponding estimation of the burden of disease. Additionally, the effects of climate and ambient air pollution on the global estimation of HAP-PM_2·5_ exposure of individuals remain unknown.

**Added value of this study**

To the best of our knowledge, the present study is the first estimation of the rural and urban as well as population exposure to HAP and associated health burden at national level. We extend the updated WHO HAP database from including 196 peer-reviewed studies (192 sample datapoints from 13 countries between 1996 and 2018) used in previous studies to the present database, which includes 282 studies (564 sample datapoints from 29 countries between 1996 and 2021). This study provides an estimation of HAP-PM_2·5_ exposure at country level, expanding the estimation of exposure from the existing six Global Burden of Diseases, Injuries, and Risk Factors Study (GBD) regions to 12 GBD regions. Also, for the first time, the inclusion of ambient air pollution (PM_2·5_) and heating degree days, as a proxy for climate, for urban and rural settings, are used in the predictive Bayesian model to obtain a more realistic estimation of the HAP-PM_2·5_ exposures. Ambient air pollution affects the exposure through air leakage or infiltration into the cooking or heating area; however, the heating degree days indicate the duration and extent of the burning of polluting fuel for heating (particularly during the winter season). Although previous studies estimate the HAP-PM_2·5_ exposure for users of different fuels and for different urban and rural settings, the present study provides an estimation of the HAP-PM_2·5_ personal exposure and indoor concentration at the national level (population exposure) across urban and rural settings. The estimated population HAP-PM_2·5_ exposure and associated attributable death rate (per 100 000 population) at national level provide new information about the contribution of household air pollution for cooking and heating to overall emissions of air pollutants and resulting health burden across various geographies (in urban and rural settings).

**Implications of all the available evidence**

This study provides important new information about the variations of HAP-PM_2·5_ personal exposure and indoor concentration across various geographies (urban and rural), as well as for users of different fuel types and stove technologies in a country. Using the estimated HAP-PM_2·5_ exposures to assess the attributable premature death further illustrates potential global health and climate co-benefits of reducing HAP by switching to clean fuels. Our evidence-based study also shows that exposure is much greater in rural than urban settings. Thus, switching to clean fuels not only reduces exposure to HAP but also diminishes the urban–rural health inequalities. The results suggest that policy interventions are needed to increase the proportion of the world population with access to clean fuels and efficient technologies to achieve the goal of universal energy access. Such policy interventions would also reduce health inequities and contribute to mitigate global climate change.

Using polluting fuels and inefficient technologies for cooking and heating results in various adverse effects on health (eg, chronic and acute ailments and premature mortality), on the environment (eg, forest degradation and deforestation), and on the climate (eg, increasing emissions of greenhouse gases and black carbon).^3^ WHO estimate the burden of household air pollution (HAP) on health contributed to about 3·2 million deaths per year in 2020.^4^ Furthermore, HAP contributes to ambient (outdoor) air pollution,^5^ which was estimated to cause 4·2 million premature deaths worldwide in 2016.^6^ Although there have been several studies on quantifying the health effects of ambient air pollution,^7^, ^8^, ^9^ estimating the effects of exposure on HAP due to cooking at the global scale^10^ and the associated health burden^4^, ^11^, ^12^, ^13^, ^14^ have received less attention, partly because of scarcity of detailed and extensive monitoring data on household air pollution. However, Bayesian methods make it possible to consider the unequal geographical representation of existing HAP-PM_2·5_ monitoring data and provide an opportunity to estimate HAP-PM_2·5_ for regions or countries with sparse or even no measurement data (appendix pp 3–4).^15^, ^16^

The first aim of this paper is to estimate levels of household exposure to PM_2·5_ air pollution (including both outdoor and indoor sources) across global settings for different urban and rural populations. Bayesian hierarchical HAP-PM_2·5_ exposure models were used to estimate annual average HAP-PM_2·5_ personal exposures (for 71 countries) and indoor concentrations (for 89 countries) first for users of different fuel types (biomass, charcoal, coal, gas, and electricity) and different stove technologies (traditional and improved) in rural and urban settings separately and then for national levels (population weighted). The Bayesian models were applied to global country data for prediction of HAP-PM_2·5_ exposures. Using a comparative risk assessment approach, the second aim is to estimate the national-level household air pollution-attributable premature mortality in urban and rural settings (data on 62 countries for personal exposure and 69 countries for indoor concentration).

## Methods

### Data source and pre-processing (sample data and prediction data)

Sample data of HAP-PM_2·5_ personal exposure and indoor concentration were obtained from an updated version of the WHO Global HAP database.^17^, ^18^ The WHO HAP database was updated from 196 publications (from the years 1996–2018), as used in previous studies,^18^ to include 282 publications (with publications from 2018 to 2021 added). The final sample included 564 datapoints (249 datapoints of personal exposure from 19 countries; 315 datapoints of indoor concentration from 29 countries) updated from 192 datapoints (140 kitchen exposures; 52 female exposures; total 13 countries) of previous studies.^10^ The number of households varies between studies and locations (country, urban, and rural settings) and ranges from three to 787. Out of 21 regions defined by gross domestic product, HAP-PM_2·5_ personal exposure studies were conducted in nine and indoor concentration in 12 regions (appendix p 6).

**Figure 1:**
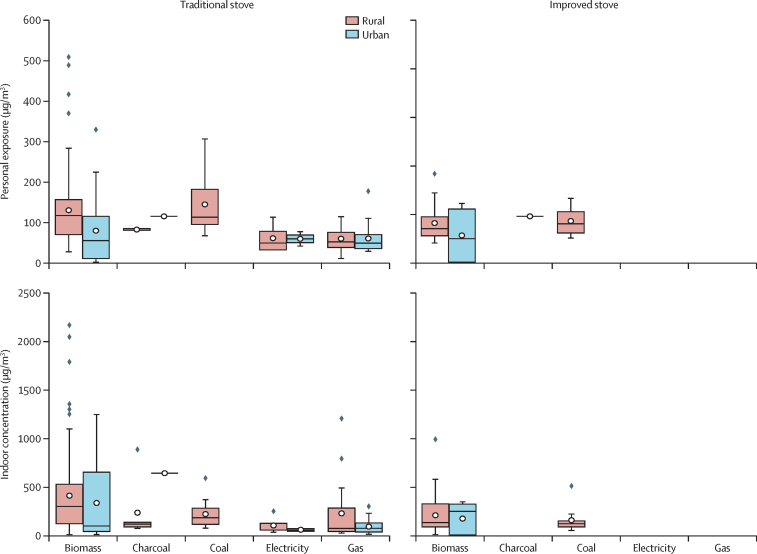
The HAP-PM_2·5_ personal exposure and indoor concentration of sample data for users of different primary fuel types and primary stove technologies (traditional and improved) in urban and rural settings HAP-PM_2·5_ data are in μg/m^3^; sample data source has been modified from the WHO Global HAP database.^17^, ^18^ HAP=household air pollution.

The exposure data for different primary fuel types (and primary stove technologies) were separated for the HAP-PM_2·5_ indoor concentration and the HAP-PM_2·5_ personal exposure (figure 1). Personal exposure refers to the air pollution that an individual is exposed to as measured by a personal air-quality monitoring device worn by the person. The measurements of HAP-PM_2·5_ exposure are obtained for at least 24 consecutive hours of day-to-day activities for about 98% of sample data (to avoid bias introduced by sampling only during cooking activities), which include the burning of household fuels both for cooking and heating (appendix p 4). The monitoring device captures pollution from outdoor cooking activities, if any, and also therefore some ambient (outdoor) air pollution. Indoor concentration refers to the time-averaged concentration of PM_2·5_ inside a house. The pollution is measured by a static air pollution monitoring device, which is placed inside the house. The placement can be in the kitchen, the living room, or elsewhere in the house where either cooking and heating, or both, take place. The stove technology was classified as traditional (unvented, no chimney in the kitchen or heating area) and improved cookstoves (vented and mentioned explicitly in the studies from which sample data were collected). The details of sample data for model development can be found in the appendix (pp 4–9).

### Summary of HAP-PM_2·5_ personal exposure sample data

Biomass fuel has the highest percentage (76%) among all fuels (ie, biomass is the most common fuel monitored across the studies that provided the data) in the personal exposure sample data for both urban and rural settings. Gas (12%) is the second highest, followed by coal (8%), charcoal (2%), and electricity (2%). Across the sample data, the average measured 24 h HAP-PM_2·5_ personal exposure (figure 1) for users of biomass fuel with traditional stoves in rural settings is 130 μg/m^3^ (range 26–508) and in urban settings 80 μg/m^3^ (1–329). For users of biomass fuel with improved stoves, the average in rural settings is 98 μg/m^3^ (48–220) and in urban setting is 68 μg/m^3^ (1–148). The average measured 24 h HAP-PM_2·5_ personal exposure for users of coal fuel in rural settings with traditional stoves is 144 μg/m^3^ (66–307) and with improved stoves 104 μg/m^3^ (60–161). There is no measurement data for users of coal fuel in urban settings. The average measured 24 h HAP-PM_2·5_ personal exposures for users of gas with traditional stoves in rural and urban settings are both 60 μg/m^3^ (10–115 for rural and 28–177 for urban). There are no measured data available for the 24 h HAP-PM_2·5_ personal exposures and indoor concentration for users of gas and electricity with improved stoves in both urban and rural settings. The average 24 h HAP-PM_2·5_ personal exposure aggregated for the WHO regions can be found in the appendix (p 12).

### Summary of HAP-PM_2·5_ indoor concentration sample data

Biomass is the highest (71%) of fuels in the indoor concentration sample data (ie, biomass is the most common fuel monitored across the studies that provided the data) for both urban and rural settings. Gas (16%) is the second highest, followed by coal (8%), electricity (3%), and charcoal (2%). The average measured 24 h HAP-PM_2·5_ indoor concentration (figure 1) for users of biomass with traditional stoves in rural setting is 408 μg/m^3^ (range 1–2170) and in urban settings 333 μg/m^3^ (2–1250). As for the biomass with improved stoves, the average in rural settings is 205 μg/m^3^ (1–990) and in urban settings 171 μg/m^3^ (1–349). The measured 24 h HAP-PM_2·5_ indoor concentrations for users of charcoal with traditional stoves are limited in urban and rural settings with no data available for users with improved stoves. The average measured 24 h HAP-PM_2·5_ indoor concentration for users of coal fuel in rural settings with traditional stoves is 218 μg/m^3^ (range 68–589) and with improved stoves 154 μg/m^3^ (43–508 μg/m^3^). The average measured 24 h HAP-PM_2·5_ indoor concentration for users of gas with traditional **s**toves is 225 μg/m^3^ in rural settings and 88 μg/m^3^ in urban settings (range for rural settings 16–1205 μg/m^3^ and for urban settings 5–297 μg/m^3^). Our sample data show relatively high average value and the higher range of values for gas in rural settings, particularly in rural settings in several countries, including Peru, Guatemala, and China. The high values can be due to the fuel stacking practice which is more common in rural households than in urban households and emissions from traditional polluting cooking fuels (as secondary fuel or stove type) perhaps mixed with exposure to fine particles. The high values can also be related to other factors (eg, tobacco smoking, lack of ventilation, and infiltration of ambient [outdoor] air). In both cases, no sample data are available.

### Predictor variable definitions

Predictor variables were selected from the above sample data for separate Bayesian models for HAP-PM_2·5_ personal exposure and indoor concentration. Five types of fuel were used in the model development, namely biomass, charcoal, coal, gas, and electricity. The two stove technologies (traditional stove and improved stove) for urban and rural settings were analysed separately. The measurement periods were grouped based on winter, summer, or whole year, assuming two seasons (winter and summer) for all countries. In addition to factors such as fuel types and stove technologies, education index and gross national income per capita, indicating the socioeconomic status at country level, were also included. Then urban and rural population-weighted ambient air pollution and heating degree days, used as proxy for climate, were added as extra predictors to the sample data. Although the pollution is primarily due to indoor burning of fuels for cooking and heating, some pollution can be attributable to infiltration of ambient (outdoor) air. This is the reason for including ambient air pollution as one of the main predictors. For detailed lists of predictor variables for personal exposure and indoor concentration see the appendix (pp 10–11). To predict HAP-PM_2·5_ exposure for countries with unknown personal exposure and indoor concentration, the same variables were collected separately for personal exposure for countries in nine GBD regions (71 countries) and indoor concentration for countries in 12 GBD regions (89 countries). The description and the list of prediction data can be found in the appendix (pp 13–14).

### Bayesian hierarchical model development

Separate Bayesian hierarchical models (for personal exposure and indoor concentration) were developed to generate accurate HAP-PM_2·5_ exposure coefficients from the sample data. These models make it possible to consider the influences of clustered data (ie, households nested in countries nested in regions) and their interactions. HAP-PM_2·5_ exposure measurements (dependent variable) were log-transformed since the distributions were right-skewed; ensuring an approximately normal distribution of the dependent variable, which improves the performance of model fitting and the prediction. Given the variations in the sample size (number of households) for a given average PM_2·5_ exposure in different studies, the log-PM_2·5_ concentrations were weighted by the number of measurements in each country. Furthermore, some of the non-categorical variables (gross national income per capita, population-weighted heating degree days, and outdoor PM_2·5_) were scaled (ie, each variable is divided by a scaling factor) and mean-centred (ie, the average of the variable is subtracted from the data) so that the continuous predictors are in a similar range. This ensures that the criterion for finding linear combinations of the predictors is based on how much variation they explain and therefore improves the numerical stability. We also excluded some 4% of the original data, namely those with significant outliers.

The Bayesian hierarchical models were implemented using the brms package in R.^16^ Nine variant models were tested including different predictors. All the models have the same baseline random-effect structure (random intercept only, at both country level and GBD regions level) in order to be consistent with the multiple nested data structure. The null model, model 0, includes all predictors with random intercept only. The other eight models have different predictors and different random slopes. The random slopes are modelled at a country level and are selected based on two steps: first, the model without random slopes (random intercept only) is fitted, second, fixed-effect predictors with the highest posterior variance are selected and included in the random-effects structure, and the model fitted again. To assess the model performance, we apply leave-one-out cross-validation to approximate the posterior predictive performance criterion using Pareto smoothed important sampling technique (appendix pp 18–20). The model with the lowest leave-one-out–posterior predictive performance criterion is retained as the best model. Three metrics for model diagnostics are used to verify whether the chosen model is a suitable model (appendix pp 21–22). The hierarchical model resulted in a Bayesian *R*^2^ of 0·67 for personal exposure, and *R*^2^ of 0·71 for indoor concentration. The fixed-effect posterior distribution statistics from the models including model coefficients, standard error, and upper and lower 95% CI can be found in the appendix (pp 23–24).

### Prediction of HAP-PM_2·5_ personal exposure and indoor concentration

HAP-PM_2·5_ exposures were estimated for countries with unknown exposure by combining the updated WHO database with the developed Bayesian hierarchical models, which (1) include quantitative past information about the monitoring data (eg, fuel specific types and stove technologies) and (2) yield probability distributions for the fitted model parameters with 95% CIs. Predicted exposures were obtained by using only the fixed-effect coefficients estimated on the sample data which represents the means of the predictive probability distributions. The annual average HAP-PM_2·5_ personal exposure and indoor concentrations are predicted for users of different fuel types (biomass, charcoal, coal, electricity, and gas) and stove technologies (traditional and improved stove) who live in urban and rural settings in each country. Average 24 h HAP-PM_2·5_ personal exposure and indoor concentrations are also predicted for the summer and winter seasons for all the studied countries.

### Attributable death rate (per 100 000 population) estimation

Attributable premature deaths due to HAP-PM_2·5_ personal exposure and indoor concentration in each country (separately for urban and rural populations) were estimated using a comparative risk assessment approach. The estimated PM_2·5_ indoor concentrations (not the PM_2·5_ personal exposure) are multiplied by a factor of 0·6 to approximate a time-averaged indoor exposure, assuming that, on average, 60% of the time is spent indoors at home (appendix p 28). For each exposure, the population attributable fraction is estimated as (RR – 1)/RR where RR is relative risk of mortality at the given exposure level. The RRs were based on the GBD 2019 meta-regression–Bayesian regularised trimmed (MR-BRT) model for the following five causes of death: ischaemic heart disease, stroke, lower respiratory infections, lung cancer, and chronic obstructive pulmonary disease.^19^, ^20^ The MR-BRTs were obtained from the GBD's public release site.^21^ For ischaemic heart disease and stroke the MR-BRT models are age specific; for other outcomes we applied the functions only at age 25 years and older, except for lower respiratory infections which we applied to all ages. We used 1000 draws of the MR-BRT curves for each disease and age group (where age specific) and normalised the RRs by the RR at the theoretical minimum risk exposure level (taken from 1000 corresponding draws, average 4·15 μg/m^3^), setting RR of 1 at PM_2·5_ exposures below the theoretical minimum risk exposure level. To estimate the attributable premature mortality for each cause, the estimated population-attributable fractions were applied to national estimates of cause-specific mortality for males and females from the 2019 GBD study^22^ per 100 000 population using population data from the GBD study.

### National-level exposure and attributable death rate estimation

The estimated HAP-PM_2·5_ exposures and attributable death rates are for typical users of each fuel type, stove technologies, and separately for urban or rural settings. To estimate exposures and death rates at the national level, three different weighted averages are used. (1) The proportion of people using each fuel type as primary fuel (biomass, charcoal, coal, gas, and electricity) in each country and in urban and rural settings is obtained from WHO for the year 2020^22^, ^23^ to weight the annual average HAP-PM_2·5_ exposures for individuals. (2) The proportion of people using each stove type, as primary stove in each country and for urban and rural settings based on the sample data and for countries within the WHO regions. The same proportion was used for all countries within each WHO region. (3) The proportion of people living in urban and rural areas in each country from the Global Human Settlement Layer.

### Role of the funding source

The funders of the study had no role in study design, data collection, data analysis, data interpretation, the writing of the report, or in the decision to submit the paper for publication.

## Results

The results for the annual average HAP-PM_2·5_ personal exposure (71 countries) and indoor concentration (89 countries) and related attributable death rate (per 100 000 population) are presented below. To explore further, the regional variations of HAP-PM_2·5_ exposure, that is, the GBD region-specific HAP-PM_2·5_ personal exposure and indoor concentration (average and 95% CIs), are also estimated and presented below. The national-level HAP-PM_2·5_ personal exposure and indoor concentration and related attributable death rate results are also presented.

**Figure 2:**
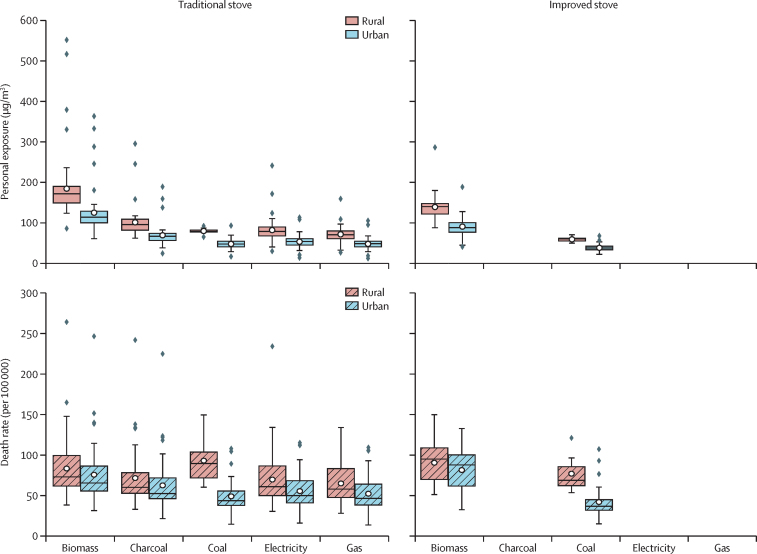
The predicted annual average HAP-PM_2·5_ personal exposure and related attributable premature death rate (per 100 000 population) for users of different fuel types and traditional and improved stove technologies in urban and rural settings HAP-PM_2·5_ data are in μg/m^3^. HAP=household air pollution.

Annual average HAP-PM_2·5_ personal exposure for 71 countries is shown in figure 2. For biomass, the annual global mean personal exposure is 184 μg/m^3^ (95% CI 166–202) for traditional stoves and 139 μg/m^3^ (126–151) for improved stoves in rural settings, and 125 μg/m^3^ (111–138) for traditional stoves and 90 μg/m^3^ (78–102) for improved stoves in urban settings. For charcoal, the annual global mean personal exposure for traditional stoves is 101 μg/m^3^ (91–111) for rural settings and 69 μg/m^3^ (62–76) for urban settings. There is no estimation for charcoal with improved stoves in rural and urban settings due to insufficient data. For coal, the annual global mean personal exposure for traditional stoves is 80 μg/m^3^ (74–85) and 59 μg/m^3^ (52–65) for improved stoves in rural settings and 49 μg/m^3^ (45–52) for traditional stoves and 38 μg/m^3^ (36–41) for improved stoves in urban settings. For clean fuels, gas and electricity, the global mean personal exposure is estimated at 71 μg/m^3^ (67–75) for gas and 82 μg/m^3^ (75–88) for electricity with traditional stoves in rural settings and 48 μg/m^3^ (45–52) for gas and 53 μg/m^3^ (50–58) for electricity with traditional stoves in urban settings. The exposure results aggregate for five WHO regions are preseted in the appendix (p 26).

Attributable premature mortality due to HAP-PM_2·5_ personal exposure (figure 2) for users of biomass with traditional stoves yields 84 deaths per 100 000 (95% CI 75–92) in rural settings and 76 deaths (67–84) in urban settings. Personal exposure for charcoal with traditional stoves results in 72 deaths per 100 000 (63–81) in rural settings and 63 deaths (54–71) in urban settings. Personal exposure for coal with traditional stoves yields 93 deaths per 100 000 (73–113) in rural settings (compared with improved stoves 77 [99–55]) and 49 deaths (44–55) in urban settings (compared with improved stoves 42 [48–37]). For traditional stoves, the personal exposure for gas and electricity results in 65 (59–71) and 70 (62–77) deaths per 100 000 in rural settings compared with 53 (47–58) and 56 (50–61) deaths in urban settings. The estimated mortality rates for users of biomass with improved stoves is slightly higher than for users of traditional stoves despite the former having lower exposure. This is because the biomass fuel with traditional stoves is not used in all the studied countries (appendix p 15); for those countries where both stove types are used for a given fuel, the estimated mortality rates for users of traditional stoves are higher than for users of improved stoves. This indicates that improved biomass stoves are less harmful to health than traditional stoves. Furthermore, our data show that users of biomass with traditional stoves are predominantly in Africa (appendix p 15) where, in general, the populations are young and therefore with comparatively low baseline mortality rates. Users of improved stoves are mainly in Asia and South America (appendix p 15), where the populations are somewhat older and thus with higher baseline mortality rates than in Africa. The death rate results aggregate for five WHO regions are preseted in the appendix (p 27).

**Figure 3:**
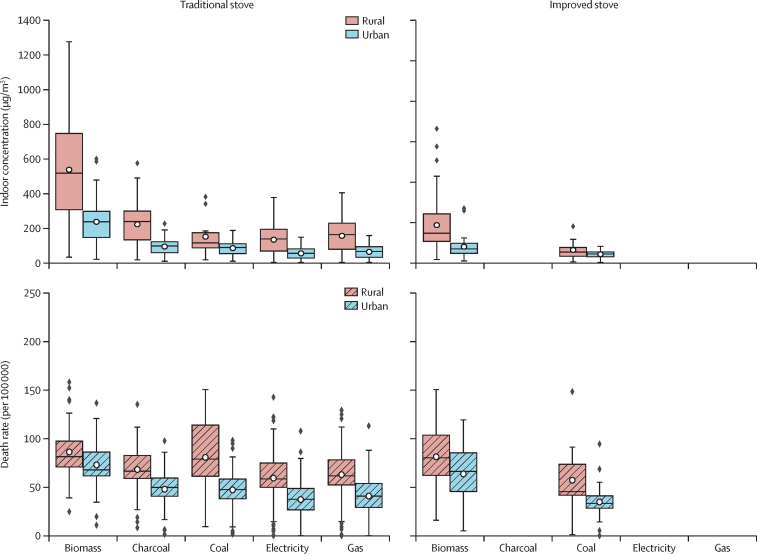
The predicted annual average HAP-PM_2·5_ indoor concentration and related attributable premature death rate (per 100 000 population) for users of different fuel types and traditional and improved stove types in urban and rural settings HAP-PM_2·5_ data are in μg/m^3^. HAP=household air pollution.

Annual average HAP-PM_2·5_ indoor concentration was estimated for 89 countries (figure 3). For biomass, the annual global mean indoor concentration is 536 μg/m^3^ (95% CI 463–608) for traditional stoves and 226 μg/m^3^ (168–283) for improved stoves in rural settings and 235 μg/m^3^ (204–266) for traditional stoves and 97 μg/m^3^ (71–123) for improved stoves in urban settings. For charcoal, the annual global mean indoor concentration for traditional stoves is 221 μg/m^3^ (188–253) in rural and 92 μg/m^3^ (79–106) in urban settings. For coal, the annual global mean indoor concentrations are 149 μg/m^3^ (70–227) for traditional stoves and 77 μg/m^3^ (26–129) for improved stoves in rural settings and 83 μg/m^3^ (72–94) for traditional stoves and 52 μg/m^3^ (45–58) for improved stoves in urban settings. For clean fuels, the indoor concentration is 154 μg/m^3^ (134–174) for gas and 132 μg/m^3^ (114–149) for electricity with traditional stoves in rural settings and 62 μg/m^3^ (53–70) for gas and 53 μg/m^3^ (45–60) for electricity with traditional stoves in urban settings. The exposure results aggregate for five WHO regions are presented in the appendix (p 28).

Assuming, on average, that households spend 60% of their time indoors (multiplied the indoor exposure by 0·6), we estimate attributable premature mortality due to HAP-PM_2·5_ indoor concentration (always per 100 000 population) for a fuel type, stove type, and separately for urban and rural settings. HAP-PM_2·5_ indoor concentration for biomass with traditional stoves results in 86 (95% CI 80–92) deaths in rural settings (compared with 81 for improved stoves [92–71]) and 73 (67–79) deaths in urban settings (compared with 64 for improved stoves [75–53]). Indoor concentration for charcoal with traditional stoves yields 68 (62–75) deaths in rural settings and 48 (43–53) deaths in urban settings. Indoor concentration for coal with traditional stoves results in 81 (51–110) deaths in rural settings (compared with 57 for improved stoves [91–23]) and 47 (42–53) deaths in urban settings (compared with 35 for improved stoves [40–30]). For traditional stoves, the indoor concentration for gas and electricity results in 65 (59–71) and 59 (53–66) deaths in rural settings and 41 (36–46) and 37 (33–42) deaths in urban settings (appendix pp 21–22). The death rate results aggregate for five WHO regions are presented in the appendix (p 29)

Estimated annual average region-specific HAP-PM_2·5_ personal exposure and indoor concentration based on country-level estimates, and their lower and upper 95% CI, are obtained by parametric bootstrapping and least-square methods for each region. For regions with skewed predicted HAP-PM_2·5_ parametric bootstrapping is used rather than a standard least-square method to avoid introducing bias in the lower bounds of confidence intervals. The results are shown in the table. The results show that biomass with traditional stoves gives rise to the highest HAP-PM_2·5_ exposures, in both rural and urban settings, while gas and electricity provide the lowest exposure. The highest HAP-PM_2·5_ exposures (personal exposure and indoor concentration) are for biomass in traditional stoves in the region of eastern sub-Saharan Africa, followed by that of western sub-Saharan Africa. In eastern sub-Saharan Africa the average personal exposure reaches 178 μg/m^3^ for urban settings and 333 μg/m^3^ for rural settings, while the indoor concentrations reach 423 μg/m^3^ for urban settings and 1075 μg/m^3^ for rural settings. The values for eastern sub-Saharan Africa are exclusively for traditional stoves; values for improved stoves are not available in this region. Eastern sub-Saharan Africa also has the highest HAP-PM_2·5_ exposure (personal exposure and indoor concentration) for users of gas and electricity with traditional stoves. This high exposure can be partly due to poor ventilation and partly to the general low quality of cooking equipment, and household characteristics. The HAP-PM_2·5_ exposures (personal exposure and indoor concentration) for western sub-Saharan Africa are the second highest among the regions. For example, the indoor concentration for users of biomass with traditional stoves reaches 242 μg/m^3^ in urban settings and 674 μg/m^3^ in rural settings. In contrast to eastern sub-Saharan Africa, western sub-Saharan Africa also has biomass for improved stoves; the exposures for improved stoves are lower than those for traditional stoves, namely 159 μg/m^3^ (urban settings) and 604 μg/m^3^ (rural settings) for indoor concentrations.

**Table:** Annual average of HAP-PM_2·5_ personal exposure and HAP-PM_2·5_ indoor concentration for each GBD region for urban and rural settings by fuel type

	**Stove technology**	**Average HAP-PM_2·5_ personal exposure (95% CI), μg/m^3^**		**Average HAP-PM_2·5_ indoor concentration (95% CI), μg/m^3^**	
		Urban	Rural	Urban	Rural
**Andean Latin America**					
Biomass	Improved stove	63 (60–65)	117 (115–120)	127 (124–129)	237 (235–239)
Biomass	Traditional	96 (93–98)	179 (176–181)	193 (190–195)	361 (358–363)
Charcoal	Traditional	62 (60–65)	116 (114–119)	125 (123–128)	235 (232–237)
Coal	Traditional	40 (37–42)	NA	80 (78–83)	NA
Electricity	Traditional	35 (32–37)	65 (62–67)	43 (25–61)	77 (48–107)
Gas	Traditional	36 (33–38)	67 (64–69)	51 (33–70)	103 (59–146)
**Central Latin America**					
Biomass	Improved stove	80 (74–84)	128 (122–134)	92 61–127)	206 (135–277)
Biomass	Traditional	110 (102–119)	170 (162–177)	184 (86–280)	400 (207–595)
Charcoal	Traditional	61 (53–69)	92 (83–101)	69 (22–116)	174 (49–299)
Coal	Improved stove	42 (NA–NA)	58 (NA–NA)	40 (NA–NA)	76 (NA–NA)
Coal	Traditional	45 (41–49)	76 (NA–NA)	62 (59–65)	116 (113–118)
Electricity	Traditional	48 (42–54)	76 (71–80)	54 (52–57)	101 (99–104)
Gas	Traditional	45 (40–50)	70 (65–75)	55 (53–58)	104 (101–106)
**East Asia**					
Biomass	Improved stove	62 (58–64)	116 (113–118)	125 (NA–NA)	234 (232–236)
Biomass	Traditional	94 (92–98)	176 (174–179)	191 (NA–NA)	357 (354–359)
Charcoal	Traditional	61 (NA–NA)	115 (NA–NA)	NA	NA
Coal	Improved stove	26 (23–28)	48 (46–51)	52 (50–56)	98 (95–100)
Coal	Traditional	39 (37–42)	74 (71–76)	79 (77–82)	149 (146–151)
Electricity	Traditional	34 (32–37)	64 (62–67)	69 (67–72)	129 (127–132)
Gas	Traditional	35 (33–37)	66 (63–68)	71 (68–73)	133 (130–135)
**Eastern sub-Saharan Africa**					
Biomass	Traditional	178 (175–180)	333 (331–335)	423 (317–529)	1075 (791–1365)
Charcoal	Traditional	115 (113–118)	216 (214–219)	181 (116–244)	465 (268–659)
Coal	Improved stove	49 (46–51)	NA	78 (62–95)	NA
Coal	Traditional	74 (72–76)	NA	141 (116–167)	NA
Electricity	Traditional	64 (62–67)	121 (119–123)	103 (76–130)	258 (180–337)
Gas	Traditional	66 (64–68)	121 (114–126)	120 (89–150)	307 (218–399)
**High-income Asia-Pacific**					
Biomass	Improved stove	NA	84 (81–86)	NA	169 (166–171)
Biomass	Traditional	NA	127 (124–130)	NA	257 (254–259)
Charcoal	Traditional	44 (41–47)	83 (80–85)	89 (87–92)	167 (164–169)
Coal	Improved stove	19 (16–21)	35 (32–37)	38 (35–40)	70 (68–73)
Coal	Traditional	28 (26–31)	53 (50–56)	57 (55–60)	107 (194–110)
Electricity	Traditional	25 (22–27)	46 (44–49)	50 (47–52)	93 (91–96)
Gas	Traditional	25 (23–28)	47 (45–50)	51 (48–54)	96 (93–98)
**High-income North America**					
Biomass	Improved stove	··	··	NA	23 (18–29)
Biomass	Traditional	··	··	NA	43 (33–53)
Coal	Improved stove	··	··	5 (5–5)	13 (12–13)
Coal	Traditional	··	··	7 (NA–NA)	20 (NA–NA)
Electricity	Traditional	··	··	4 (3–5)	12 (9–15)
Gas	Traditional	··	··	5 (4–6)	14 (10–18)
**North Africa and Middle East**					
Biomass	Improved stove	··	··	110 (85–146)	438 (199–614)
Biomass	Traditional	··	··	189 (119–257)	678 (344–1080)
Charcoal	Traditional	··	··	NA	NA
Coal	Improved stove	··	··	35 (27–45)	115 (29–198)
Coal	Traditional	··	··	60 (50–70)	188 (97–277)
Electricity	Traditional	··	··	57 (32–82)	129 (64–175)
Gas	Traditional	··	··	68 (44–92)	162 (73–237)
**South Asia**					
Biomass	Improved stove	69 (67–71)	129 (127–132)	178 (166–190)	503 (455–551)
Biomass	Traditional	105 (103–107)	197 (195–199)	203 (180–228)	564 (498–633)
Coal	Traditional	43 (41–46)	82 (80–84)	116 (108–125)	366 (299–434)
Electricity	Traditional	38 (36–41)	71 (69–74)	81 (72–90)	237 (200–275)
Gas	Traditional	39 (37–42)	73 (71–76)	90 (83–98)	272 (216–331)
**Southeast Asia**					
Biomass	Improved stove	97 (91–102)	139 (131–147)	112 (110–115)	210 (207–212)
Biomass	Traditional	134 (119–150)	192 (172–212)	171 (168–173)	319 (317–322)
Charcoal	Traditional	69 (66–72)	102 (98–107)	111 (108–113)	208 (205–210)
Coal	Improved stove	39 (38–41)	55 (52–58)	47 (44–49)	87 (85–90)
Coal	Traditional	51 (49–53)	73 (70–77)	71 (69–74)	133 (131–136)
Electricity	Traditional	55 (52–59)	86 (79–94)	62 (59–64)	116 (113–118)
Gas	Traditional	50 (46–54)	75 (69–82)	63 (61–66)	119 (116–121)
**Southern Latin America**					
Biomass	Improved stove	54 (52–57)	150 (144–154)	109 (107–112)	204 (202–207)
Biomass	Traditional	82 (80–85)	196 (189–203)	166 (164–169)	312 (309–314)
Charcoal	Traditional	54 (51–56)	114 (109–118)	108 (106–111)	202 (200–205)
Coal	Improved stove	23 (20–25)	70 (NA–NA)	46 (43–48)	85 (NA–NA)
Coal	Traditional	34 (32–37)	92 (NA–NA)	69 (67–72)	130 (NA–NA)
Electricity	Traditional	30 (27–32)	98 (94–102)	60 (58–63)	113 (111–116)
Gas	Traditional	30 (28–33)	87 (84–89)	62 (59–64)	116 (113–118)
**Tropical Latin America**					
Biomass	Improved stove	··	··	58 (57–58)	133 (133–134)
Biomass	Traditional	··	··	112 (100–122)	258 (237–279)
Charcoal	Traditional	··	··	57 (53–59)	106 (103–109)
Coal	Traditional	··	··	36 (NA–NA)	NA
Electricity	Traditional	··	··	31 (29–34)	59 (56–62)
Gas	Traditional	··	··	32 (29–35)	61 (58–63)
**Western sub-Saharan Africa**					
Biomass	Improved stove	79 (NA–NA)	148 (NA–NA)	159 (NA–NA)	604 (NA–NA)
Biomass	Traditional	120 (118–122)	225 (222–227)	242 (240–245)	674 (626–723)
Charcoal	Traditional	78 (75–80)	146 (143–149)	157 (155–160)	254 (221–287)
Coal	Improved stove	33 (30–35)	NA	66 (64–69)	NA
Coal	Traditional	50 (48–52)	NA	101 (98–103)	NA
Electricity	Traditional	44 (41–46)	82 (79–84)	88 (85–90)	180 (158–202)

Data are n (95% CI). Personal exposure values are not estimated for three regions, namely high-income North America, Africa and the Middle East, and Tropical Latin America due to insufficient sample data. GBD=Global Burden of Diseases, Injuries, and Risk Factors Study. HAP=household air pollution. HAP=household air pollution. NA=data not available.

As for other regions, north Africa and the Middle East also have high indoor concentration values. For biomass and traditional stoves, the indoor concentrations reach 189 μg/m^3^ in urban settings and 678 μg/m^3^ in rural settings. The rural values are higher than those for western sub-Sahara but considerably lower than those for eastern sub-Sahara. As in all the regions, the indoor concentration values for north Africa and the Middle East are much lower for improved stoves, namely 110 μg/m^3^ (urban settings) and 438 μg/m^3^ (rural settings). For north Africa and Middle East, the prediction is only available for HAP-PM_2·5_ indoor concentration because of lack of sample data for personal exposure.

**Figure 4:**
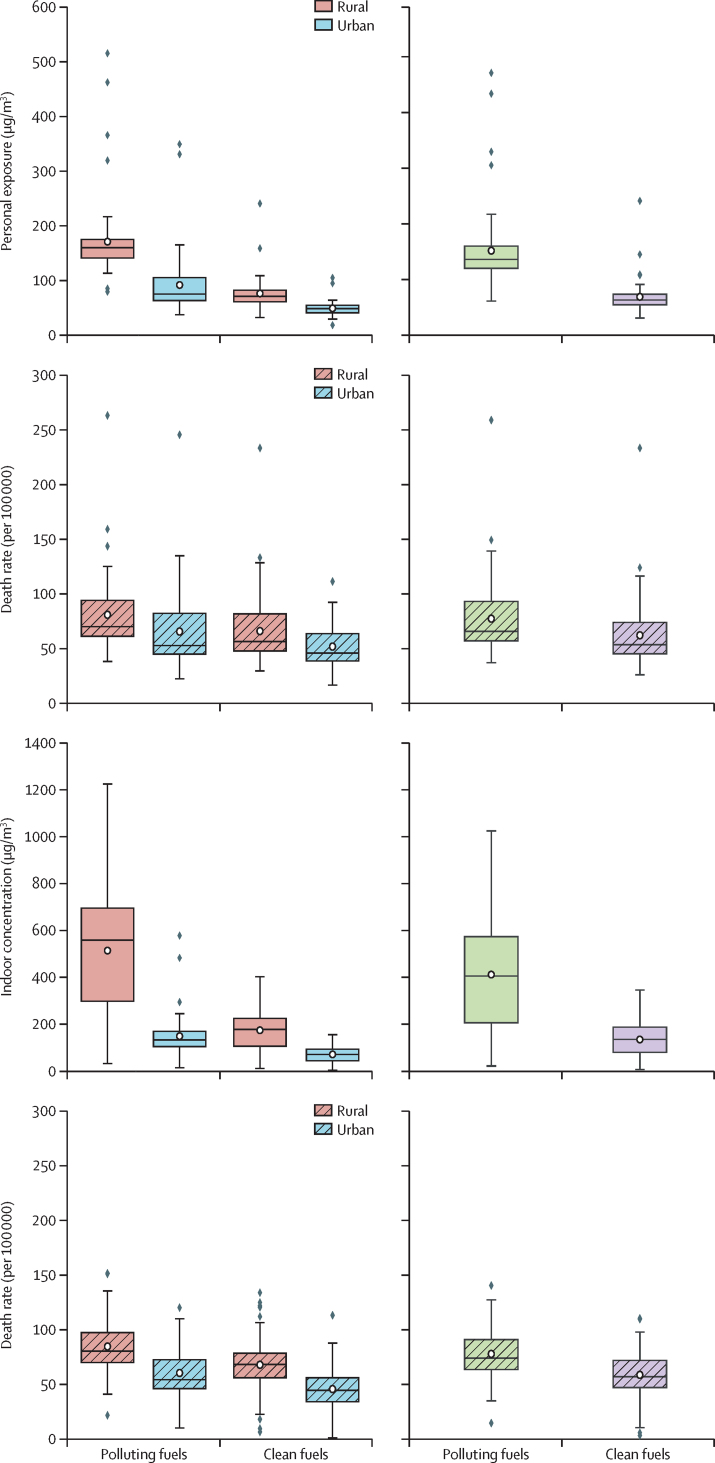
The estimated urban, rural (left-hand plots) and national-level (right-hand plots) annual weighted average HAP-PM_2·5_ personal exposure, HAP-PM_2·5_ indoor concentration, and related attributable premature death rate (per 100 000 population) of polluting solid fuels and clean fuels HAP-PM_2·5_ data are in μg/m^3^. The national-level results aggregated for five WHO regions can be found in the appendix (pp 34–37). HAP=household air pollution.

The urban, rural, and national-level annual weighted average HAP-PM_2·5_ personal exposure for 62 countries (nine GBD regions) and HAP-PM_2·5_ indoor concentration for 69 countries (12 GBD regions), as well as related attributable premature death rates of polluting solid fuels and clean fuels have been estimated (figure 4).

The national-level HAP-PM_2·5_ results show there is a large difference in the exposure level of polluting solid fuels and clean fuels and associated health burden between urban and rural settings (figure 4). The estimated HAP-PM_2·5_ personal exposure of 62 countries shows that the use of polluting solid fuels for cooking and heating in 2020 led to a national-level 24 h average exposure of 151 μg/m^3^ (95% CI 133–169), with a rural household average of 171 μg/m^3^ (153–189) and an urban average of 92 μg/m^3^ (77–106). The use of clean fuels gives rise to a national-level average exposure of 69 μg/m^3^ (62–76), with a rural average of 76 μg/m^3^ (69–83) and an urban average of 49 μg/m^3^ (46–53). Using the predicted PM_2·5_ personal exposure, the attributable premature mortality from the use of polluting solid fuels at the national level is, on average, 78 (69–87), with a rural average of 82 (73–90) and an urban average of 66 (57–75). However, the attributable premature mortality from the use of clean fuels at the national level is on average 62 (55–70), with a rural average of 66 (58–74) and an urban average of 52 (47–57). Mapping the national-level exposure and death rate results for 62 countries is presented in the appendix (pp 30–31).

The estimated HAP-PM_2·5_ indoor concentration of 69 countries from five WHO regions shows that the use of polluting solid fuels resulted in an average PM_2·5_ indoor concentration of 412 μg/m^3^ (95% CI 353–471), with a rural average of 514 μg/m^3^ (446–582) and an urban average of 149 μg/m^3^ (126–173). The use of clean fuels (gas and electricity), however, led to an average PM_2·5_ indoor concentration of 135 μg/m^3^ (117–153), with a rural average of 174 μg/m^3^ (154–195) and an urban average of 71 μg/m^3^ (63–80). As indicated above, all indoor concentrations are multiplied by a factor of 0·6. Using a time-averaged PM_2·5_ indoor concentration, the attributable premature death rate due to polluting fuels is, on average, 78 (72–84), with a rural average of 86 (79–92) and an urban average of 60 (54–66). The attributable premature mortality from the use of clean fuels is, on average, 59 (53–64), with a rural average of 68 (62–74) and an urban average of 45 (41–50). Mapping the national-level exposure and death rate results for 69 countries is presented in the appendix (pp 32–33).

## Discussion

The UN Sustainable Development Goal (SDG) 7 is to ensure universal access to affordable, reliable, and modern energy services by 2030. This goal requires universal access to clean fuels and technologies for cooking by 2030. Although considerable progress has been made since 2010 as to universal energy access, in agreement with SDG7, progress has been unequal across regions and between rural and urban populations. The present HAP-PM_2·5_ personal exposure and indoor concentration results show that exposures due to polluting fuels are generally much higher in rural settings than in urban settings despite urban settings having a higher ambient air pollution. Explaining the spatial variations of exposure in urban and rural settings between countries is complex and requires consideration of many factors in addition to the ambient air pollution. However, the ambient air pollution can have profound effect on the spatial variations of exposure in rural and urban settings within a country. The urban–rural disparities of HAP-PM_2·5_ exposure between countries can be partly related to inequalities in the socioeconomic status of households between rural and urban settings, and partly to housing conditions, household-building interactions such as ventilation (eg, window or door openings), and to cooking equipment, which is generally of lower quality in rural settings.

The user-level HAP-PM_2·5_ personal exposure and indoor concentration results show that HAP-PM_2·5_ exposures from traditional stoves in rural and urban settings are much higher than those from improved stoves. Thus, improved stove interventions can potentially reduce HAP-PM_2·5_ exposure, particularly in rural settings. However, implementation of improved stove technologies can be challenging, especially in many rural settings, because of sociocultural household traditions. Therefore, such interventions should have flexibility and be tailored to the needs, preferences, and specific culture of households.^24^, ^25^

Based on our global average model estimates for personal exposure, switching from biomass with traditional stoves in rural settings to biomass with improved stoves would reduce the average HAP-PM_2·5_ personal exposure by 45 μg/m^3^—ie, from 180 μg/m^3^ to 135 μg/m^3^. But switching from biomass with traditional stoves to cleaner fuels such as gas would reduce the average HAP-PM_2·5_ personal exposure by 98 μg/m^3^—ie, from 180 μg/m^3^ to 82 μg/m^3^. For the indoor concentration in rural settings, switching from biomass with traditional stoves to biomass with improved stoves would reduce the HAP-PM_2·5_ indoor concentration from 536 μg/m^3^ to 226 μg/m^3^, an average of 310 μg/m^3^, while switching from biomass with traditional stoves to cleaner fuels such as gas would reduce the indoor concentration from 536 μg/m^3^ to 154 μg/m^3^, an average of 382 μg/m^3^.

The results suggest that switching from polluting fuels (biomass, charcoal, and coal) to cleaner fuels (gas and electricity) for heating and cooking can potentially reduce the national-level HAP-PM_2·5_ personal exposure on average by 53% and the national-level HAP-PM_2·5_ indoor concentration on average by 65%. However, there is a considerable variation between rural and urban settings, partly reflecting inequality in energy access. Switching from polluting fuels for heating and cooking to cleaner fuels can reduce the HAP-PM_2·5_ personal exposure in rural settings by 54% and in urban settings by 38%. Switching from polluting fuels for heating and cooking to clean fuels can reduce the HAP-PM_2·5_ indoor concentration by 65% in rural and by 48% in urban settings.

The potential of switching to clean fuels, particularly in rural households is challenging, and is affected by income, education, household size, fuel availability, location, and other sociodemographic factors.^26^ Many studies show that fuel stacking (using multiple stove-and-fuel cooking combinations within the same household) remains prevalent in rural households.^27^ A 2022 study found that affordability (eg, the fuel price being too high), stove functionality (broken equipment), and stove and equipment incompatibility (eg, incompatibility of stove with large pots) are overwhelming drivers of fuel stacking.^28^ Thus, energy policy must offer flexibility and consider the sociocultural interests of households, to achieve the goals of universal energy access and decarbonising energy systems.

Two previous studies of the national-level 24 h average HAP-PM_2·5_ indoor concentrations due to polluting fuels for India estimate an overall national-level 24 h average kitchen concentration of 600 μg/m^3^ and 450 μg/m^3^.^10^, ^29^ For comparison, the present study estimates the average HAP-PM_2·5_ indoor concentration in India as 450 μg/m^3^ for rural and 155 μg/m^3^ for urban settings, with the overall national level as 315 μg/m^3^. The differences between the previous kitchen and the present indoor concentration estimates are partly related to different type of sample data and the weighting factors used in the present study. The present sample data derive not only from the kitchen (cooking) but also from the living room (heating) and the percentage of fuel use by population in urban and rural settings, as given by WHO.^23^ By contrast, the data in the two previous studies are exclusively from the kitchen and the data source is different, namely the India National Family and Health Survey 2015. For national-level 24 h average HAP-PM_2·5_ personal exposure in India, previous studies, which made estimates for male, female, and child exposures, yield an average between 275 μg/m^3^ and 258 μg/m^3^.^10^, ^29^ Our study, however, yields the overall national level 24 h average personal exposure for India as 137 μg/m^3^. The annual average estimated death rate (per 100 000 population) due to personal exposure at national level in India is therefore different from that of WHO;^4^ while the estimation in the current study for India is 108 (95% CI 94–121), the WHO estimation for India is 82 (CI 58–103).

The present estimates of household air pollution-induced mortality include some overlap with estimates of mortality due to ambient air pollution, which is also the case for the WHO estimates.^4^ The results indicate that the potential reduction in HAP-PM_2·5_ exposures through switching to cleaner fuel could lower the national-level HAP-PM_2·5_ personal exposure death rates (per 100 000 population) by 21% and indoor concentrations death rates by 26%. The reductions in HAP-PM_2·5_ personal exposure can reduce death rates (per 100 000 population) by 20% in rural and by 17% in urban settings. The reductions in indoor concentrations can diminish the death rate by 23% in rural and by 26% in urban settings. Our annual average estimated death rate (per 100 000 population) due to personal exposure at national level compared with those from WHO for the African region has an average difference of 21% compared with the WHO estimation.

The present study provides new global and national results on HAP-PM_2·5_ personal exposure and indoor concentration for clean and dirty primary fuels in urban and rural settings, as well as the attributable mortality rates. Predicting household air pollution, however, is complex and impacted by several factors including individual behaviour (eg, tobacco smoking status) and housing characteristics (eg, age, materials, ventilation, window opening, and infiltration of ambient [outdoor] air) which are not typically captured in the monitored data. Updating the sample data with detailed information on housing characteristics would greatly improve future predictions. Furthermore, the individual studies used for collecting the sample data contain a limited number of monitored households, so that the data might not be nationally fully representative for the rural and urban populations of the associated countries. The sample data in the WHO Global HAP database are collected using different methods (eg, different monitoring technologies) and are processed as well as classified in different ways, both of which might affect the data. The sample data used in the current study provide limited information on secondary fuel and stove types, because the data are collected for the primary fuel and stove types. There are also some limitations on the types of fuels collected in the sample data. For example, limited exposure measurements on kerosene in the WHO global HAP database prevented its inclusion in the modelling.

Despite these limitations, the Bayesian predictive models developed here make it possible to explore a wide range of PM_2·5_ exposures for countries worldwide, where the range is a function of fuel use, stove types, and urban and rural locations. The availability of monitored household air pollution data from around the world is continuously growing and improving. This growth and improvement should eventually make it possible to estimate PM_2·5_ exposure and related health effects for the regions and countries missing from this study. The inclusion of heating degree days, as a proxy for climate, plays an important role in the Bayesian exposure model development. Heating degree days provide useful information on the duration and the extent of polluting fuel burning for heating for countries with cold climate (the current study shows that the winter months have higher exposure and concentrations than the summer months). The plan is to explore heating degree days under future climate scenarios and its effect on associated exposure estimation in a further expansion of this study.

In conclusion, the present study shows that switching to clean fuels can substantially reduce HAP-PM_2·5_ exposure and, by implication, associated death rates. The estimated average HAP-PM_2·5_ personal exposure and indoor concentrations for all fuel types exceed the 35 μg/m^3^ annual average threshold recommended by WHO's Interim Target-1. The results show that there is substantial difference in exposure between rural and urban settings, highlighting the inequality in energy access and health; furthermore, that improved stove interventions and mitigation of ambient air pollution sources are needed to maximise the benefits to health. Considerable policy interventions are needed to rapidly increase the number of people with access to clean fuels and stove technologies by 2030 to achieve the goals of universal energy access and address health inequities in urban–rural settings.

## Data sharing

The final datasets are available on reasonable request to the corresponding author.

## Declaration of interests

We declare no competing interests.
