# Supplementary material for: Urban–rural disparity in global estimation of PM2·5 household air pollution and its attributable health burden
Source: Lancet Planet Health. 2023 Aug 7;7(8):e660–72. doi: 10.1016/S2542-5196(23)00133-X (PMC10958988; doi:10.1016/S2542-5196(23)00133-X)
Supplement: Supplementary appendix [file mmc1.pdf]

THE LANCET
Planetary Health

Supplementary appendix

This appendix formed part of the original submission and has been peer reviewed. We post it as supplied by the authors.

Supplement to: Mohajeri N, Hsu S-C, Milner J, et al. Urban–rural disparity in global estimation of $PM_{2·5}$ household air pollution and its attributable health burden. *Lancet Planet Health* 2023; **7:** e660–72.

Supplementary appendix

Urban-rural disparity in global estimation of $PM_{2.5}$ household air pollution and its attributable health burden

Nahid Mohajeri PhD [a,h], Shih-Che Hsu MSc[b], James Milner PhD[c], Jonathon Taylor PhD[d], Gregor Kiesewetter PhD[e], Prof Agust Gudmundsson PhD[f], Harry Kennard PhD[b,g], Prof Ian Hamilton PhD[b], Prof Mike Davies PhD[a]

[a]Institute of Environmental Design and Engineering, Bartlett School of Environment, Energy and Resources, University College London, London, UK

[b]Energy Institute, University College London, London, UK

[c]Dept of Public Health, Environments, and Society, London School of Hygiene & Tropical Medicine, London, UK

[d]Dept of Civil Engineering, Tampere University, Tampere, Finland

[e]International Institute for Applied Systems Analysis (IIASA), Laxenburg, Austria

[f]Department of Earth Sciences, Royal Holloway, University of London, Egham, UK.

[g]Center on Global Energy Policy, Columbia University, New York, NY, USA

[h]Correspondence to: Institute of Environmental Design and Engineering, Bartlett School of Environment, Energy and Resources, University College London, London, UK

Appendix

Contents

Background... 3

Data source...4

Sample data description...6

Prediction data...2

Method ……... 18

Results... 25

Background

Several studies provide estimates of the HAP-$PM_{2.5}$ exposures in rural and urban settings of different regions where different fuel types and stove technologies where considered, but these have mostly focused on a very limited number of countries. [1,2] However, there are very few studies that estimated the exposure to household air pollution at global scale, and these do not separate, for the effect of different fuel types from that of different stove types. [3] The paucity of such studies is partly due to lack of HAP-$PM_{2.5}$ monitoring data as well as insufficient information as to the type of fuels and stoves used for cooking, and heating monitoring location (kitchen, living room), housing material, ventilation, time spent near the cooking area, socio-demographic factors (such as age, gender, education) and income. Additionally, there have been no studies that include the effects of climate and ambient air pollution on the HAP-$PM_{2.5}$ exposure of individuals. This is important since urban and rural HAP-$PM_{2.5}$ exposure of individuals using the same fuels and the same stove technologies within a given country can vary partly depending on the climate as well as ambient levels of air pollution. [4]

About 3.2 million deaths were attributable to household air pollution (HAP) in the latest WHO Burden of disease (BoD) for household air pollution report. [5] Three types of information were used by WHO to estimate the health impacts of HAP. These are: (i) the proportion of households exposed to HAP from polluting fuels and technologies using the *Household Energy Database which is a collection of national survey data*, [6] (ii) personal level HAP exposure based on the *WHO Global HAP Database*, [7,8] and (iii) relative risk (RR) for a disease caused by HAP-$PM_{2.5.}$ integrated exposure response (IER) curves developed by Institute for Health Metrics and Evaluation (IHME) for the Global Burden of Disease Study. Relative risk estimated from different source of $PM_{2.5}$ exposures (outdoor air pollution, active smoking, second-hand smoke, and household air pollution. [9-12]

Since 1990, the IHME GBD has conducted annual comparative risk assessments, describing the extent and distribution of ill health globally by age, gender, and disease for various risk factors. [13] A study in 2000 was the first to include HAP as a major risk factor. [12,14] Using data on household cooking fuel types from national censuses and demographic and health surveys, the GBD conducted sex-specific meta-analyses of HAP epidemiological studies to provide male and female relative risks of developing a particular disease based on a binary indicator of whether a household used dirty or clean fuels. [14] The estimated relative risk for a particular disease was combined with the proportion of the population in each country that cooked with dirty fuels to determine global mortality due to HAP. The results of this study might misclassify risk levels of diseases as they apply a single exposure measure to all males or all females worldwide. [14] To refine the results, integrated exposure-response (IER) curves and the sex/age-specific exposures were introduced in the GBD 2010. [9,11,12] In the GBD 2015 report (Forouzanfar et al., 2015) and the GBD 2016 report (Gakidou et al., 2017), region specific and country specific HAP-PM2.5 exposures were estimated using the *WHO Global HAP Database*. [15,16] These exposures were then applied to outcome-specific exposure-response curves to estimate disease burden attributable to HAP.

While WHO and IHME GBD studies mentioned above provided country-specific estimates of the burden of disease due to HAP exposure for males and females, their estimation did not consider the variations of HAP across various geographies (urban and rural), as well as for different fuel and stove types. Furthermore, in these studies, the exposure levels obtained from the proportion of households exposed to HAP from polluting fuels rather than obtained from the HAP-$PM_{2.5}$ exposure for users of different fuel types and stove technologies.

WHO Global HAP Database contains quantitative HAP-$PM_{2.5}$ personal exposure and indoor concentration monitoring data from peer-reviewed studies updated in 2018. [8] We extend the updated *WHO Global HAP Database* from the 196 peer-reviewed studies (years 1996-2018) used in previous studies, [3] to the present base, which includes 282

studies (years 2018-2021). Using an established robust method, a Bayesian hierarchical model, developed in earlier studies, [3, 6] we expand the prediction of exposure from the previous 6 GBD regions [3] to the present 12 GBD regions. Our study complements and adds to previous work in that we differentiate between fuel types (Biomass, Coal, Charcoal, Gas, Electricity), stove technologies (Traditional and Improved), as primary fuel and stove technology, and urban and rural settings. In addition, for the first time, ambient air pollution ($PM_{2.5}$) and Heating Degree Days (HDD), the latter a proxy for climate at urban and rural settings within each county/subnational, are included in predictive models. The result is refined and improved estimation of HAP-$PM_{2.5}$ personal exposure and indoor concentration. These two variables provide information on how the use of fuels varies with the seasons within a country, as well as an indication of duration and extent of polluting fuel burning for heating.

Data source

The *World Health Organization Global HAP database* contains detailed measurements of HAP-$PM_{2.5}$ personal exposure and indoor concentration data from peer-reviewed and published studies. [8, 17] Some details include primary fuel types (e.g., dung, residues, wood, charcoal, coal, gas, electricity), primary stove technologies (traditional, improved), monitoring location (e.g., kitchen, living room, personal), household geographic locations (rural, urban), as well as measurements periods (e.g., specific months, seasons, or the whole year). In addition to the common scholarly literature search engines, such as Google Scholar and Scopus, a tool from Chan Zuckerberg Initiative, namely Meta (a Toronto-based artificial intelligence scientific literature search engine) is used to find studies relevant to the household air pollution. [18] Without relying on term matches and SEO (search engine optimization), Meta uses natural language processing (NLP) to prioritize papers that are most relevant to a research and are from commonly cited scientists. A total number of 939 studies is identified after the term ‘household air pollution’ was searched in Metadata. After the citations and the relevance of papers (recommended by Meta) were checked for two iterations, 392 studies between June 2018 and June 2021 were sifted based on the following criteria. Finally, 86 studies were selected and added on the top of the 196 existing studies. This made the total number of studies used in the current paper up to 282. The final sample included 564 data points (249 data points of personal exposure from 19 countries; 315 data points of indoor concentration from 29 countries). The data points vary for each country and depends primarily on the geographical location (x, y) of households. For example, the sample data were collected for more than 30 geographical locations across the whole China from 25 studies, and more than 22 geographical locations across the whole India from 41 studies. Also, it should be mentioned that the number of households vary for each geographical locations.

- Studies should have at least an abstract
- Inclusion of indoor monitored matters
- Exclusion of health case control study
- Exclusion of household intervention study
- Exclusion of monitoring comparison study
-

To ensure the studies include valid HAP-$PM_{2.5}$ measurements, those studies lack the following information were excluded.

- HAP-PM2.5 measurements obtained over a period of less than 24 hours (< 24 h);
  Out of 564 sample data only 9 measurement (~1.6% of sample data) includes the average exposure timing between 12 and 24 (hour <24 hour). These measurements belong to Guatemala and Honduras (Central Latin America region), and Bangladesh (South Asia region), and 4 out of 9 measurements belong to Bangladesh. As excluding these 3 countries, would affect our sample size and thus model stability (particularly for the regional variations).
- Fine particulate matter ($PM_{2.5}$) measurement (mostly arithmetic mean but also geometric mean or median value if there was no arithmetic mean is given)
- Household location from rural and urban settings

- Cooking and heating Fuels
- Stove technologies

Table S1. Out of 21 regions defined by GBD, HAP-$PM_{2.5}$ personal exposure estimations were conducted in 9 and indoor concentration in 12. HAP-$PM_{2.5}$ personal exposure and indoor concentration estimations were conducted in 5 out of 6 regions defined by WHO.

Personal Exposure and indoor concentration (WHO regions)	Personal exposure (GBD regions)	Indoor concentration (GBD regions)
African Region	Andean Latin America	Andean Latin America
Eastern Mediterranean Region	Central Latin America	Central Latin America
Regions of the Americas	East Asia	East Asia
South-East Asian Regions	Eastern Sub-Saharan Africa	Eastern Sub-Saharan Africa
Western Pacific Region	High-income Asia Pacific	High-income Asia Pacific
	South Asia	High-income North America
	Southeast Asia	North Africa and Middle East
	Southern Latin America	South Asia
	Western Sub-Saharan Africa	Southeast Asia
		Southern Latin America
		Tropical Latin America
		Western Sub-Saharan Africa

Sample data description and data processing

The sample data is collected for different primary fuel types including wood, crop residues, and dung which are combined into the category of 'biomass', LPG (Liquefied petroleum gas), biogas, and natural gas are combined into the category of 'gas'. Different primary types of stove technologies are collected (e.g., solid fuel stove, open fire stove, biomass, gas stove, improved stove) and divided into two main categories: traditional stoves and improved stoves. All the types of fuel and stove technologies were collected for different urban and rural settings where the semi-rural and peri-urban locations are grouped as 'rural' (figure S1). The measurement periods were obtained from the publications that explicitly mention the season or the month(s) of measurements. The months were classified into two seasons, namely summer (17%) and winter (29%), depending on the country location in the hemisphere. Whenever the measurements were done in mixtures of months or across several months in two seasons, the data is classified as whole year (54%).

Ambient air pollution and Heating Degree Days (HDD), used as proxy for climate, were added as extra predictors to the sample data. Average HDD data for 2000 (1985-2015) were obtained from NASA Earth Exchange Global Daily Downscaled Projections (NEX-GDDP) with a grid resolution of 25 km. [19] The grids were aggregated into urban and rural and estimated for population-weighted mean HDD. Ambient air pollution ($PM_{2.5}$) concentrations were collected from the sample studies wherever values were provided (40% of 564 samples). If ambient air pollution was missing, we used the latitude and longitude of the location where the HAP-$PM_{2.5}$ measurement took place to find the closet air pollution monitoring station using 'OpenAQ' platform. [20] 'OpenAQ' is an open-source platform which collects data from multiple sources including reference-grade and low-cost sensor data, to fill in the missing data. For the station-based data, whenever possible, we obtained the average ambient air pollution fit with the study period (winter, summer, or whole year) and for the same time for which the HAP-$PM_{2.5}$ measurements were obtained. Otherwise, an average typical year was collected.

The HAP-$PM_{2.5}$ personal exposure and indoor concentrations from the sample data for different primary fuel types and for urban and rural settings are plotted against the population-weighted mean heating degree days (HDD) and ambient air pollution ($PM_{2.5}$). The sample data follows a right-skewed distribution, as seen on the upper and right margins of figure S1a, S2a. There relations between personal exposure and HDD, on one hand (figures S1b and S1c), personal exposure and outdoor $PM_{2.5}$, on the other hand (figures S1d and S1e), are disperse, both for rural and urban settings. Similarly disperse are the relations between indoor concentrations and HDD (figures S2b and S2c) and between indoor concentrations and outdoor $PM_{2.5}$ (figures S2d and S2e), again both for rural and urban settings.

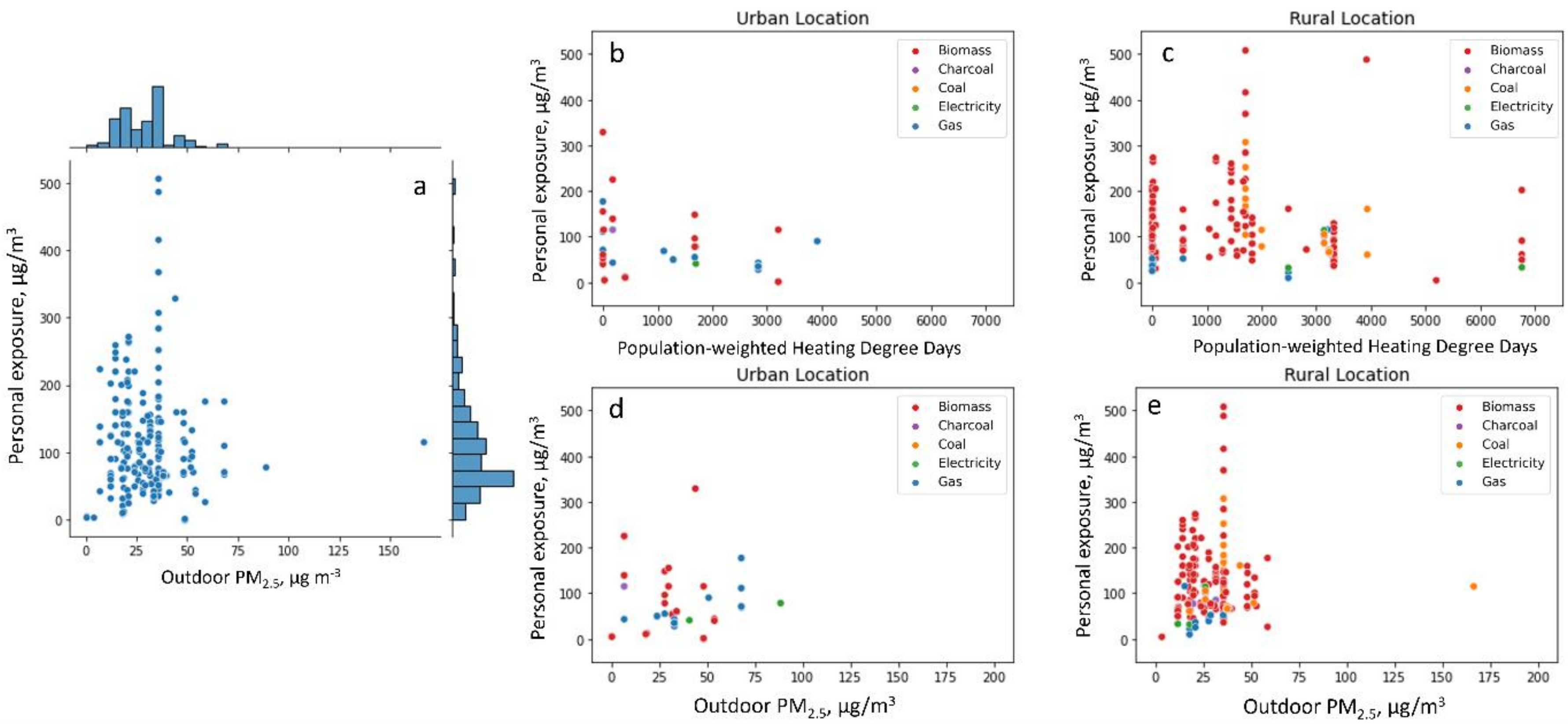

Figure S1. **(a) The HAP-PM$_{2.5}$ personal exposure, in µg/m^3, versus outdoor PM$_{2.5}$, µg/m^3, of sample data and their right-skewed distributions. HAP-PM$_{2.5}$ personal exposure, µg/m^3, of sample data versus population-weighted heating degree days separately for urban (b) and rural settings (c) and for different primary fuel types. HAP-PM$_{2.5}$ personal exposure, µg/m^3, of sample data versus outdoor PM$_{2.5}$, µg/m^3, separately for urban (d) and rural settings (e) and for different primary fuel types.**

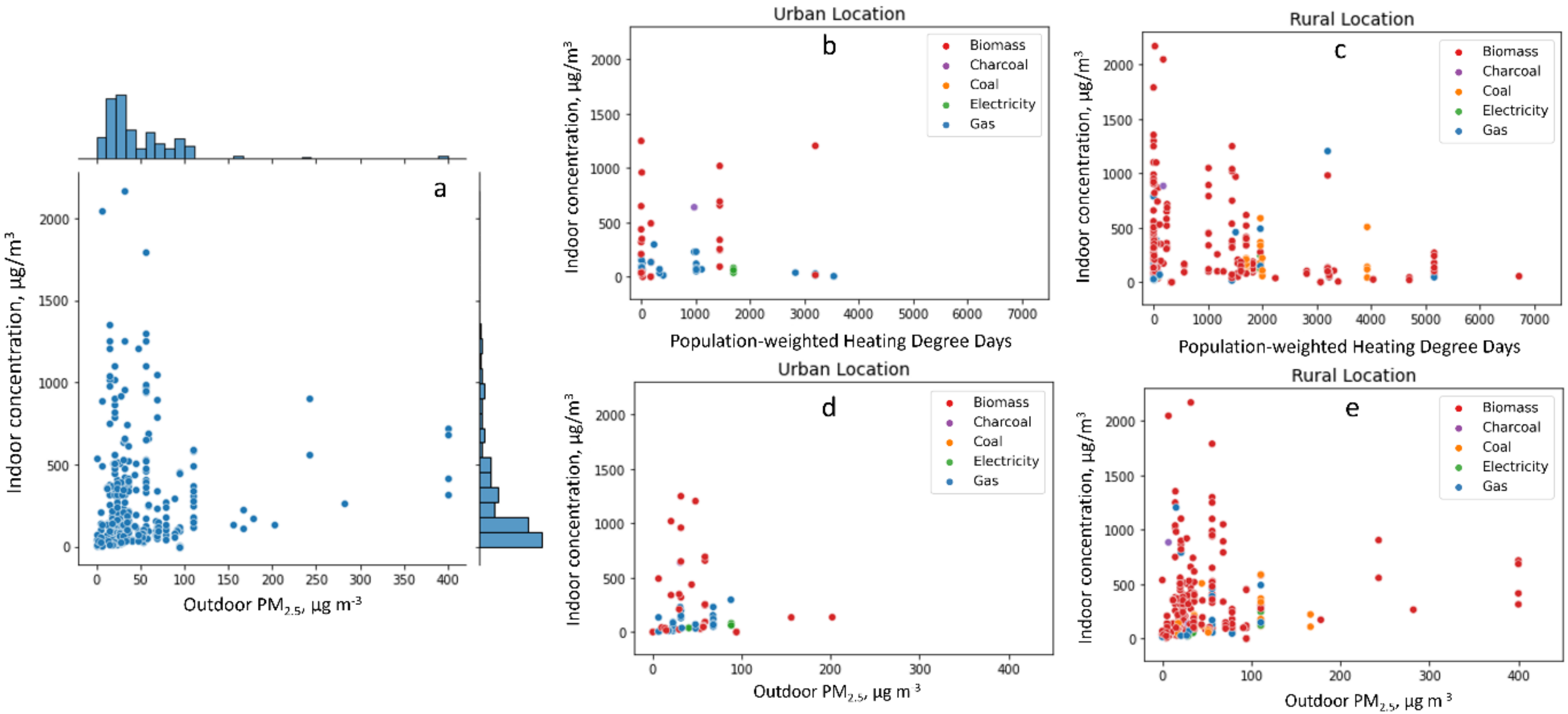

Figure S2. (a) The HAP-PM2.5 indoor concentration, μg/m^3, versus outdoor PM2.5, μg/m^3, of sample data and their distributions. HAP-PM2.5 indoor concentration, μg/m^3, of sample data versus population-weighted heating degree days separately for urban (b) and rural (c) settings and for different primary fuel types. HAP-PM2.5 indoor concentration, μg/m^3, of sample data versus outdoor PM2.5, μg/m^3, separately for urban (d) and rural € settings and for different primary fuel types.

List of main predictive variables from sample data for model development

Table S2. Characteristics and source of sample data for the HAP-$PM_{2.5}$ Personal exposure. [8, 17]

***HAP-$PM_{2.5}$* Personal exposure**			
**Data**	**Quantity**	**Resolution**	**source**
Global Burden of Disease (GBD)	9 Regions	Region	GBD
Country	19	Country	WHO
Location	Urban, Rural	Household/village/town	WHO
Primary fuel types	Biomass, Coal, Charcoal, Gas, Electricity	Household/village/town	WHO
Primary stove types	Traditional and Improved stove	Household/village/town	WHO
$PM_{2.5}$ ambient (outdoor) air pollution	Urban, Rural	Household location or closest monitoring station	WHO + OpenAQ
HDD (heating degree days)	Urban and rural population weighted	Gridded 25 km for the year 2010 HDD averages (1995-2025) – 50th percentile	NASA
Education index	19	Country	UNDP
GNI per capita	19	Country	UNDP
Season	Winter/summer/whole year	Household location	WHO
Household $PM_{2.5}$ personal exposure	249	Average household PM2.5 obtained for above 24h	WHO

Table S3. Characteristics and source of sample data for the HAP-$PM_{2.5}$ indoor concentration. [8, 17]

***HAP-$PM_{2.5}$* Indoor concentration**			
**Data**	**Quantity**	**Resolution**	**Source**
Global Burden of Disease (GBD)	12 Regions	Region	GBD
Country	29	Country	WHO
Location	Urban, Rural	Household/village/town	WHO
Primary fuel types (Heating and cooking)	Biomass, Coal, Charcoal, Gas, Electricity	Household/village/town	WHO
Primary stove types	Traditional stove Improved stove	Household/village/town	WHO
$PM_{2.5}$ ambient (outdoor) air pollution	Urban, Rural	Household location or closest monitoring station	WHO + OpenAQ
HDD (Heating Degree Days)	Urban and rural population weighted	Gridded 25 km for the year 2010 HDD averages (1995-2025) – 50th percentile	NASA
Education index	29	Country	UNDP
GNI per capita	29	Country	UNDP
Season	Winter/summer/whole year	Household location	WHO
Household $PM_{2.5}$ personal exposure	315	Average household Above 24h	WHO

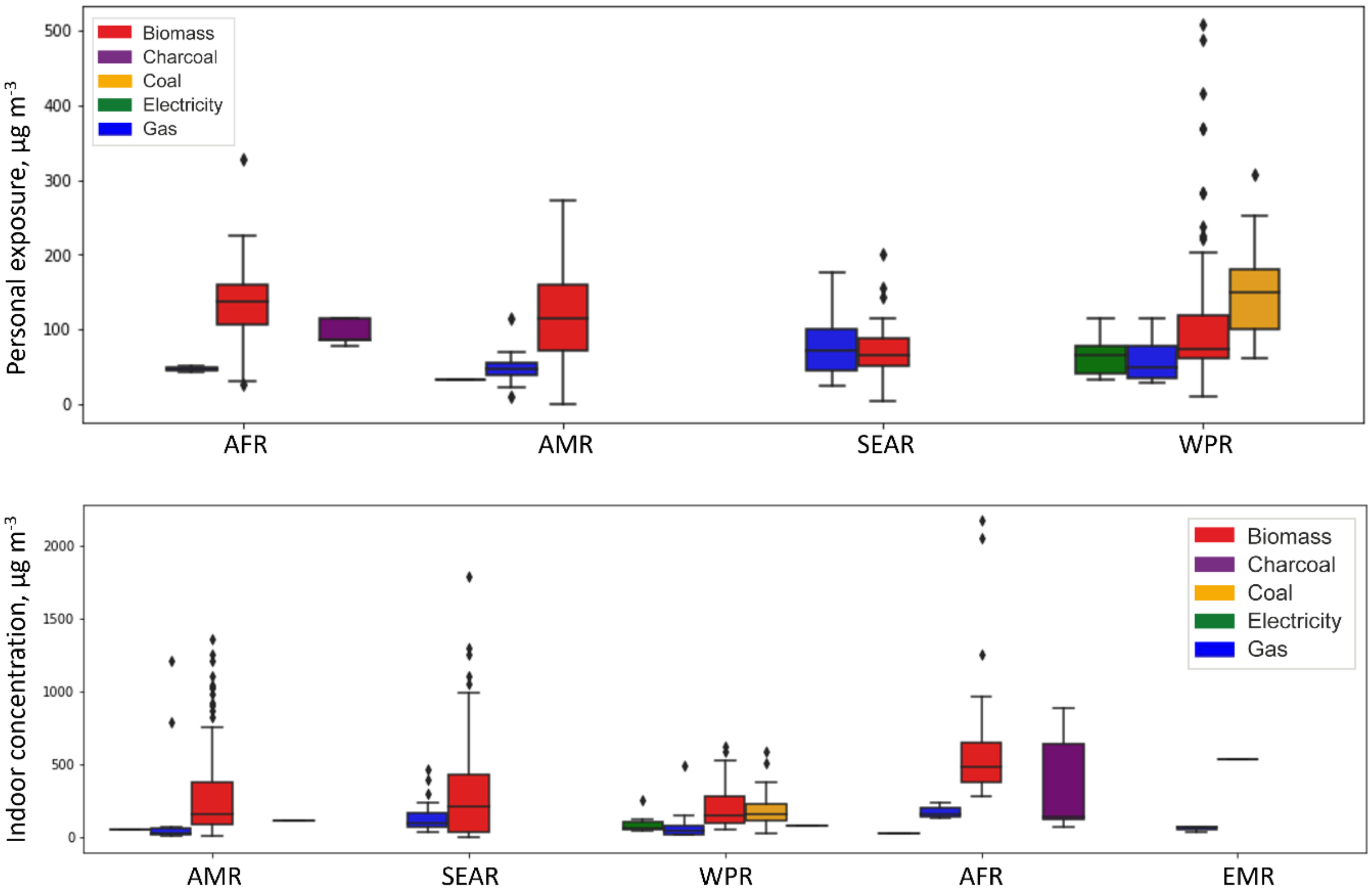

Figure S3. The average measured 24-hour HAP-$PM_{2.5}$ personal exposure and indoor concentration, µg/m^3, data aggregated for the WHO regions for different fuel types (WHO, 2018b). AFR (African Region), AMR (Region of the Americas), SEAR (South-East Asian Region), WPR (Western Pacific Region), Eastern Mediterranean Region (EMR).

Prediction data

To predict $PM_{2.5}$ personal exposure for countries in 9 GBD regions and indoor concentration for countries in 12 GBD regions (the same regions as the sample data), we collected and pre-processed the data for countries with unknown personal exposure and indoor concentration (Table S4). To have the same data structure as the sample data, the following data and pre-processing steps were used. (i) First, the types of fuel use for 71 countries for personal exposure prediction and 89 countries for indoor concentration prediction were taken from IEA World Energy Outlook 2021. [21] The fuel type data was obtained using the Greenhouse Gas and Air Pollution Interactions and Synergies (GAINS) model, an integrated assessment framework describing the pathways of atmospheric pollution from anthropogenic driving forces to relevant health and environmental impacts. [22] The model was initially developed at the International Institute for Applied Systems Analysis (IIASA). Most of the data were obtained at the country level. There are, however, sub-national level data for several countries, including China and India, as well as regional level data (e.g., for Western Africa, Eastern Africa, and Central Latin America). To apply the aggregated regional data to the countries within that region, the same types of fuels were used for the all the countries within a given region (e.g., within the regions of Western Africa, Eastern Africa, and Central Latin America). However, the sub-national data remain the same resolution since it can provide a better accuracy for the model (figure S4). (ii) Second, stove technologies were obtained from GAINS model at the same spatial resolution. [23] Types of fuels obtained from GAINS were grouped into the same categories as the sample data (biomass, charcoal, coal, gas and electricity). The same data pre-processing was done for the stove technologies to make this variable consistent with the training data. New coal/biomass stoves and fan-assisted cooking stoves are classified as an improved stove (figure S5).

Ambient $PM_{2.5}$ concentrations were also obtained from GAINS at grid level - 0.1 degree/~10km and aggregated into urban and rural population-weighted mean concentrations. [24,25] To do this, the population data are used from the Global Human Settlement Layer (GHSL) with 250 m grid resolution for 2015. [26] The data are aggregated to 0.1 degree and separated into urban and rural using polygon layer of the Global Rural-Urban Mapping Project GRUMP (v1.1). We classify population as *urban* where is located inside a polygon with a total population >100,000 and with a population density of >1000/km^2, and the rest as *rural*. We estimate the population-weighted mean ambient $PM_{2.5}$ concentrations for rural and urban areas from the grided data described above (figure S6, S7). Education index (UNDP, 2020) and GNI per capita (UNDP, 2020) are two variables that were also included. [27] To convert the predicted exposures at the level of individuals to the national level, the proportion of population with primary reliance on fuels for cooking by fuel type (%) for the year 2020 is used, as obtained from WHO. [6, 28] Baseline mortality data were obtained from GBD national estimates for both males and females. Meta-regression–Bayesian regularized trimmed (MRBRT) functions for attributable premature mortality were obtained from GBD2019 for five different causes of mortality (chronic obstructive pulmonary disease, ischemic heart disease, lower respiratory infections, stroke, and lung cancer).

Table S4. Characteristics and source of prediction data (countries with unknown exposure) for the HAP-$PM_{2.5}$ personal exposure and indoor concentration

Predicted data

**Data**	**Quantity**	**Resolution**	**Source**
Global Burden of Disease (GBD) regions	9 region (personal exposure)<br>12 region (indoor concentration)	Region	GBD
Country and sub-national	71 countries (personal exposure)<br>89 (indoor concentration)	Country and subnational	WHO
Location	Urban, Rural	Country and subnational	GAINS
Fuel types<br>(Heating and cooking)	Biomass, Coal, Charcoal,<br>Gas, Electricity	Country and subnational	GAINS
Stove types	Traditional stove<br>Improved stove	Country and subnational	GAINS
$PM_{2.5}$ ambient (outdoor) air pollution	Urban, Rural<br>population weighted	Gridded 0.1 degree<br>Average for country and subnational	GAINS
HDD (Heating Degree Days)	Urban and rural<br>population weighted	Gridded 25 km for the year 2010<br>HDD averages (1995-2025) – 50th percentile	NASA
Education index	71 countries (personal exposure)<br>89 (indoor concentration)	Country	UNDP
GNI per capita	71 countries (personal exposure)<br>89 (indoor concentration)	Country	UNDP
Season	Winter/summer/whole year	Country and subnational	WHO

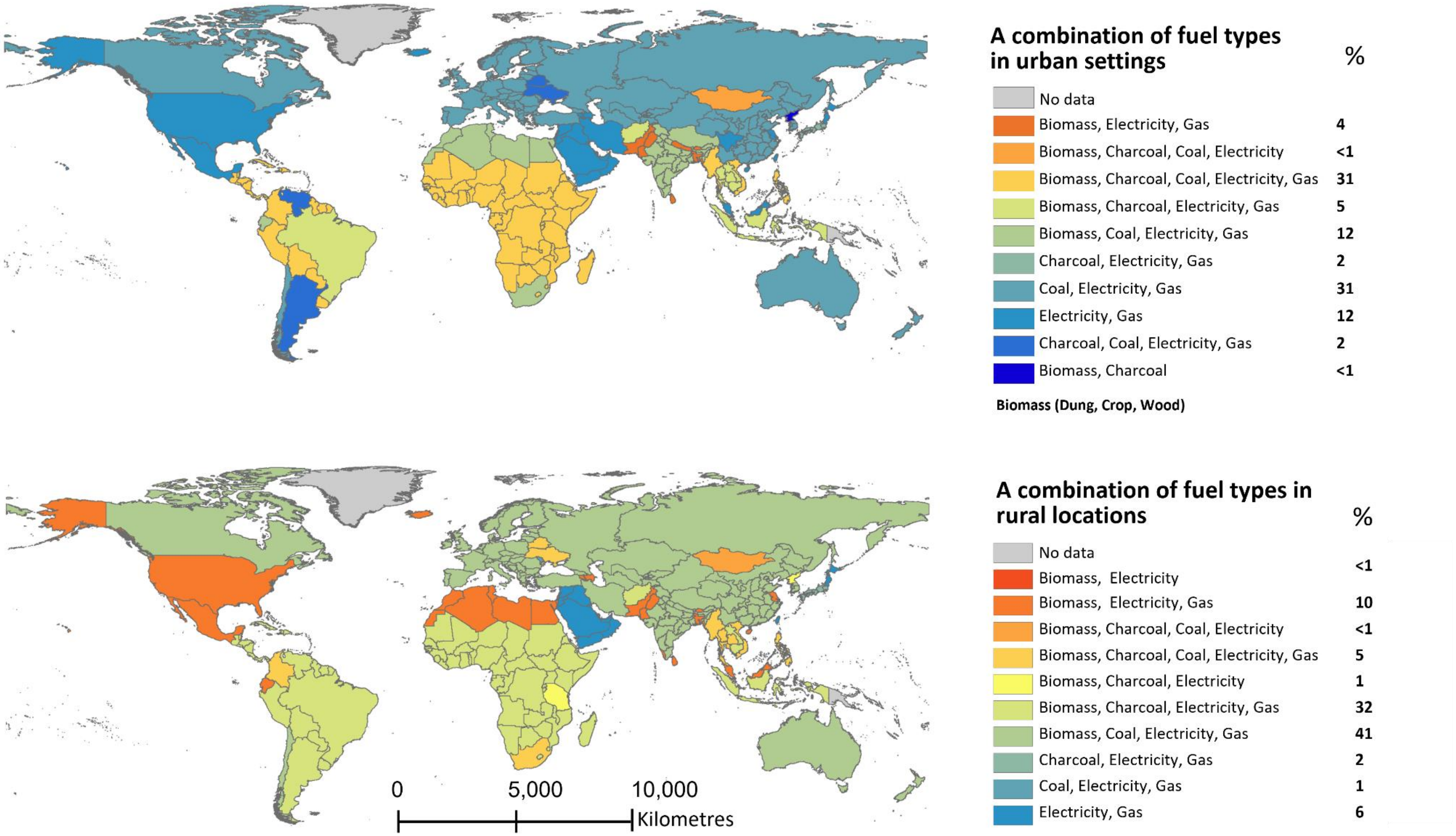

Figure S4. Percentage of fuel type combinations at country level and sub-national level in urban and rural settings. [21]

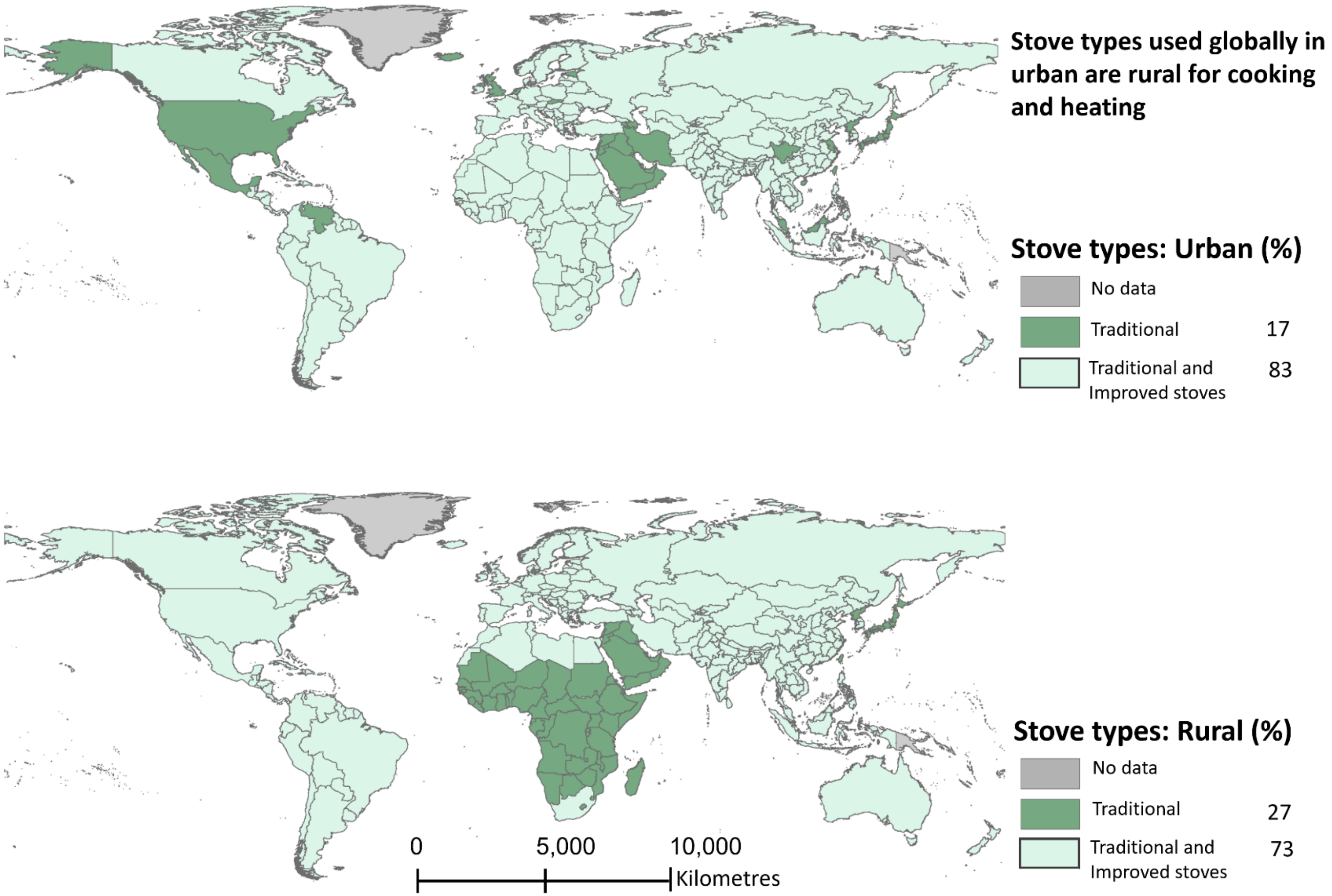

Figure S5. Percentage of stove technology (traditional and improved stove) combinations at country level and sub-national level in urban and rural settings. [23]

The following equation is used to estimate the population-weighted mean ambient $PM_{2.5}$. [24, 25, 29]

$$\sum (populastion_{ik} \times concentration_{ik}) / \sum population_{ik}$$

Where '*I*' is the urban or rural settings, *population* $_{ik}$ is the population in the grid cell k and, *concentration* $_{ik}$ is the concentration in the grid cell k.

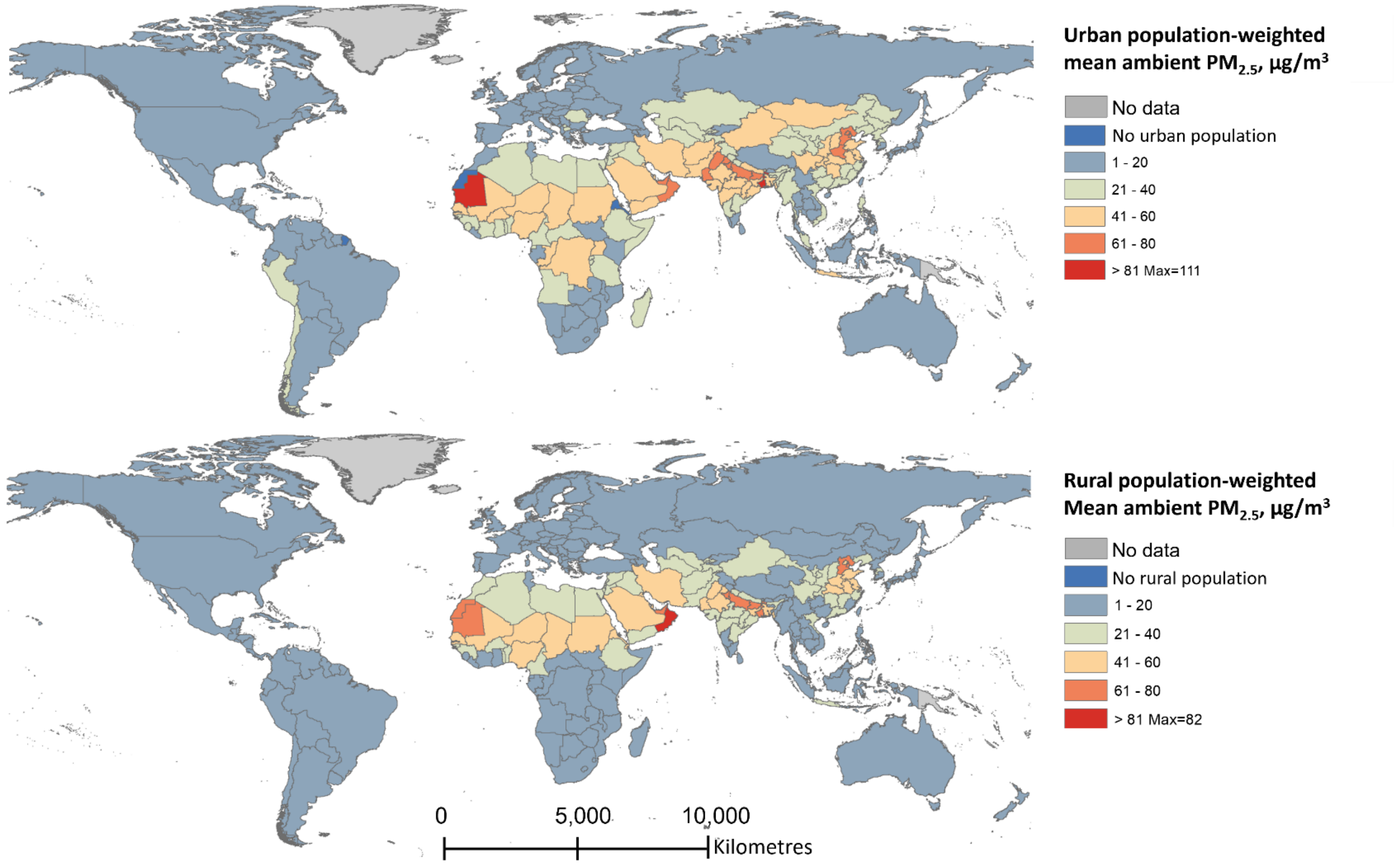

Figure S6. Population-weighted mean ambient $PM_{2.5}$, µg/m^3 at country level and sub-national level in urban and rural settings.

The following equation is used to estimate the population-weighted mean Heating Degree Days.[30] Where R_i is the ratio of the population in grid i to a reference population (e.g. Country), HDD_j is unweighted heating degree-day value for the grid I, and N is total number of grids in the country.

$$HDD = \sum_{i=1}^{N} (R_i \times HDD_i)$$

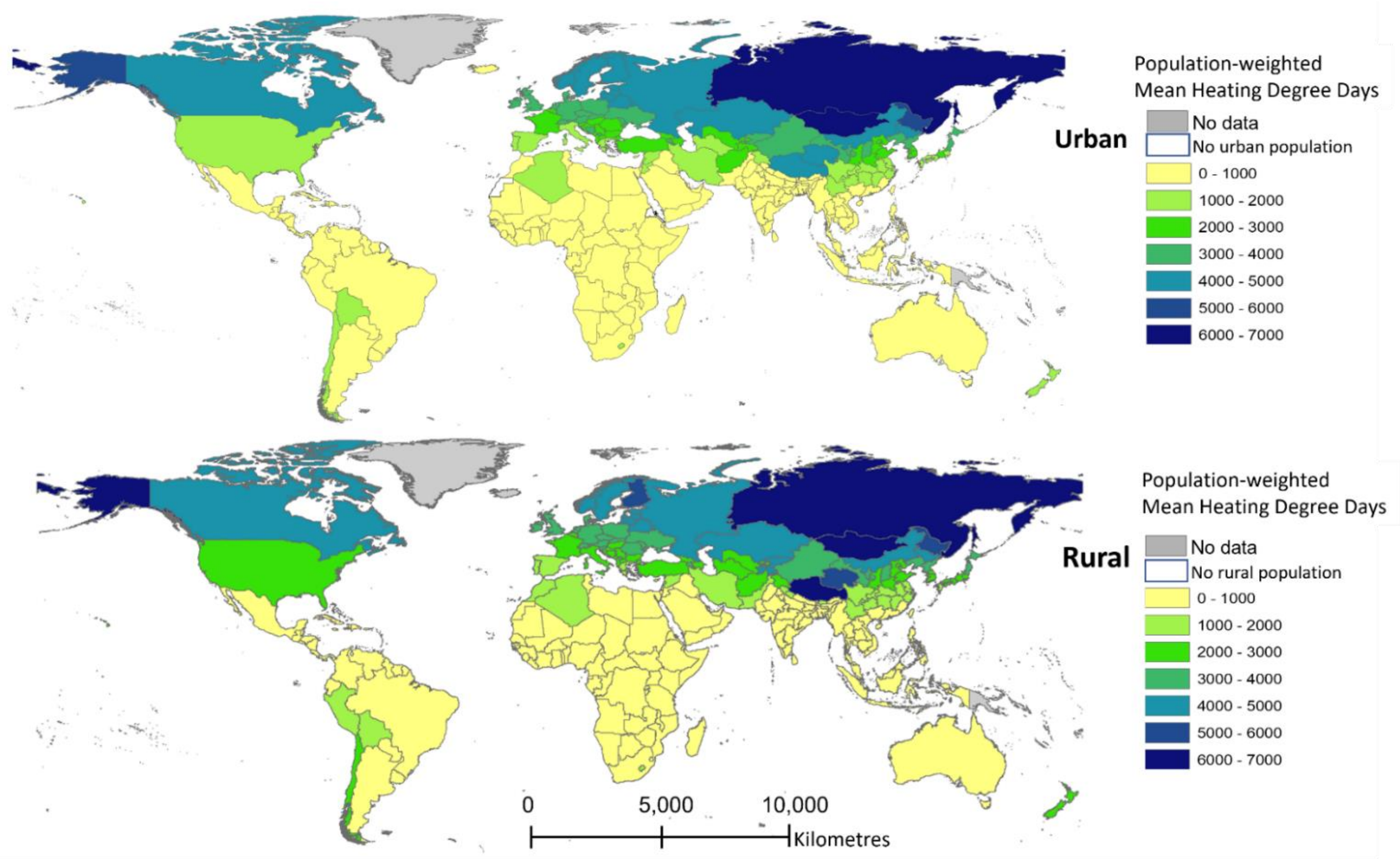

Figure S7. Population-weighted mean Heating Degree Days at country level and sub-national level in urban and rural settings.

Method

Bayesian hierarchical model development

All developed models were fitted with the same procedure and hyperparameter tunning. A weakly informative prior with a Cauchy (0, 1), centre 0 and scale 1, as prior distribution, was set for estimating the regression co-efficient. [31, 32] To sample posterior distributions for parameter estimation, the No-U-Turn Sampler (NUTS) is applied, that is, an extension to Hamiltonian Monte Carlo (HMC) algorithm, which is a Markov chain Monte Carlo (MCMC) algorithm using Stan language in *brms* package. The sampler is run for 3600 iterations, with a burn-in of 600 samples, for 3 chains, to obtain a joint posterior density of 3000 × 3 samples. The chains of the sampler are parallelised on 3 CPU cores. The adapt_delta parameter (i.e., target acceptance probability) which is a tuning parameter in the NUTS sampler for Hamiltonian Monte Carlo, is set at 0.95 to ensure the model convergence.

Model selection and checking

We used Leave-One-Out (LOO) Cross-Validation to estimate pointwise out-of-sample prediction accuracy from each fitted Bayesian model. [33] Pareto Smoothed Important Sampling technique is used to approximate the ELPD_LOO (expected log pointwise predictive density) and its standard error (SE_ELPD_LOO). The model with the (absolute) lowest ELPD_LOO is retained as the best model. The Leave-One-Out (LOO) package in '*R*' provides different evaluation metrics, including LOOIC (Leave-One-Out Information Criterion) and its standard error (SE_LOOIC). The model with the lowest LOOIC is retained as the best model. ELPD_DIFF which is the difference in ELPD_LOO for two models and its standard error (SE_DIFF) are also computed (details can be found in Table S6, S7).

Three metrics for model diagnostics are used to test if the chosen model is a suitable model. The metrics are: (i) Posterior Predictive Check (PPC), using visual inspection, to verify whether simulated data distribution resembles the true data distribution; (ii) the Bayesian R^2; and (iii) visualisation of the distribution of the posterior residuals to ensure the assumption of normally distributed residual is fulfilled. The first PPC (figure 8A) shows several simulated data distributions (one for each iteration) against the true data distribution ($y_{predicted}$ vs $y_{measurement}$). The second PPC (figure 8B) shows the distribution of the mean computed on the predicted results by the model against the true mean of the dependent variable. The hierarchical model incorporates several variables including fuel types (biomass, charcoal, coal, gas, electricity), stove types (traditional, improved) for different urban and rural settings, resulted in a Bayesian R^2 of 0.67 (standard error: 0.007; Q2.5 (lower quartile) = 0.65; Q97.5 (upper quartile) = 0.68) for personal exposure (µg/m^3), and R^2 of 0.71 (standard error: 0.007; Q2.5 (lower quartile) = 0.70; Q97.5 (upper quartile) = 0.73) for indoor concentration (µg/m^3). The posterior residuals are also shown (figure 8 C) to ensure the assumption of normally distributed residuals is fulfilled. Thus, model checking indicates that the selected models ('Model 3' and 'Model 4') are optimal among the assumed models. The inclusion of ambient air pollution and HDD are quite important determinant of the model and affect the model selection and model performance. The fixed-effect posterior distribution statistics from the model (coefficients, standard error, upper and lower 95% credible intervals) are shown in the figure S10 and Tables S8, S9.

Table S5. list of nine considered models for both HAP-$PM_{2.5}$ personal exposure and indoor concentration for evaluation to select the best model. The bold models, 'Model 3' is selected as the best model for HAP-$PM_{2.5}$ personal exposure and 'Model 4' is selected as the best model for HAP-$PM_{2.5}$ indoor concentration.

Model No.	Definitions
**Model 0**	Includes all predictors (see Table 3 for details) with random intercept only.
**Model 1**	Model 0 (except 'Education Index' as random slope since it is the only fixed effect with high variance) and a random slope for 'HDD', 'Outdoor PM2.5', 'Fuel Type' and 'Stove Technology'
**Model 2**	Model 0 and a random slope for 'Fuel Type' and 'Stove Technology'
**Model 3**	**Model 0 and a random slope for 'HDD', 'Outdoor PM2.5', 'Fuel Type' and 'Stove Technology' and 'Urban/Rural Location'.**
**Model 4**	**Model 0 without 'Education Index' and a random slope for 'HDD', 'Outdoor PM2.5', 'Fuel Type' and 'Stove Technology'.**
**Model 5**	Model 0 without 'Education Index' and 'Season' and a random slope for 'HDD', 'Outdoor PM2.5', 'Fuel Type' and 'Stove Technology'.
**Model 6**	Model 0 without 'Education Index', 'Season', and 'GNI per capita' and a random slope for 'HDD', 'Outdoor PM2.5', 'Fuel Type' and 'Stove Technology'.
**Model 7**	Model 0 without 'Education Index' and 'GNI per capita' and a random slope for 'HDD', 'Outdoor PM2.5', 'Fuel Type' and 'Stove Technology'.
**Model 8**	Model 1 without 'Urban/Rural Location' and 'Season'

Table S6. Model selection criteria for HAP-PM2.5 personal exposure. Model 3 is selected as the best predictive model.

MODEL NO	ELPD_DIFF	SE_DIFF	ELPD_LOO	SE_ELPD_LOO	LOOIC	SE_LOOIC
MODEL 3	0	0	-3811.977	855.7	7624	1711.5
MODEL 4	-67	66.1	-3879	856.3	7758	1712.6
MODEL 7	-73	64.1	-3885	859.9	7771	1719.9
MODEL 1	-78	65.6	-3891	861.7	7782	1723.4
MODEL 2	-1810	431.4	-5623	818.2	11245	1636.5
MODEL 0	-2264	429.4	-6076	728.1	12153	1456.1
MODEL 6	-3572	580.4	-7385	1031.2	14770	2062.3
MODEL 5	-3576	581.1	-7388	1035.1	14776	2070.1
MODEL 8	-3654	586.3	-7466	1022.6	14933	2045.2

Table S7. Model selection criteria for HAP-PM2.5 indoor concentration. 'Model 4' is selected as the best predictive model.

MODEL NO	ELPD_DIFF	SE_DIFF	ELPD_LOO	SE_ELPD_LOO	P_LOO	SE_P_LOO	LOOIC	SE_LOOIC
**M4**	**0**	**0**	**-11928.48**	**790.2015**	**1324.697**	**147.1833**	23856.96	1580.403
M3	-0.490906	113.4571	-11928.97	797.4028	1480.518	169.83843	23857.94	1594.806
M7	-22.2992	12.93799	-11950.78	793.879	1341.097	150.68929	23901.55	1587.758
M1	-29.20403	12.634	-11957.68	794.8654	1357.34	155.3682	23915.36	1589.731
M6	-96.09468	115.4392	-12024.57	804.1409	1238.245	141.30736	24049.15	1608.282
M5	-100.9502	116.052	-12029.43	805.4518	1245.126	145.18841	24058.86	1610.904
M2	-505.632	199.429	-12434.11	751.7897	1135.55	117.07899	24868.22	1503.579
M8	-569.7285	298.4588	-12498.21	850.0376	1149.358	133.192	24996.41	1700.075
M0	-607.7766	247.299	-12536.25	729.0475	801.417	79.48478	25072.51	1458.095

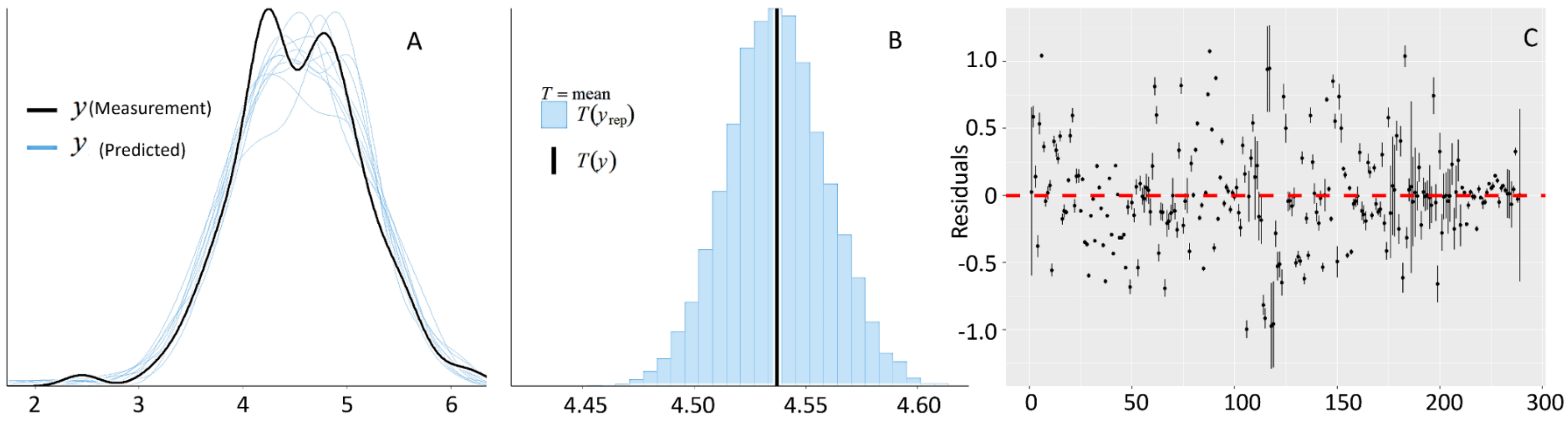

Figure S8. Posterior Predictive Check (PPC) for HAP-PM2.5 personal exposure model

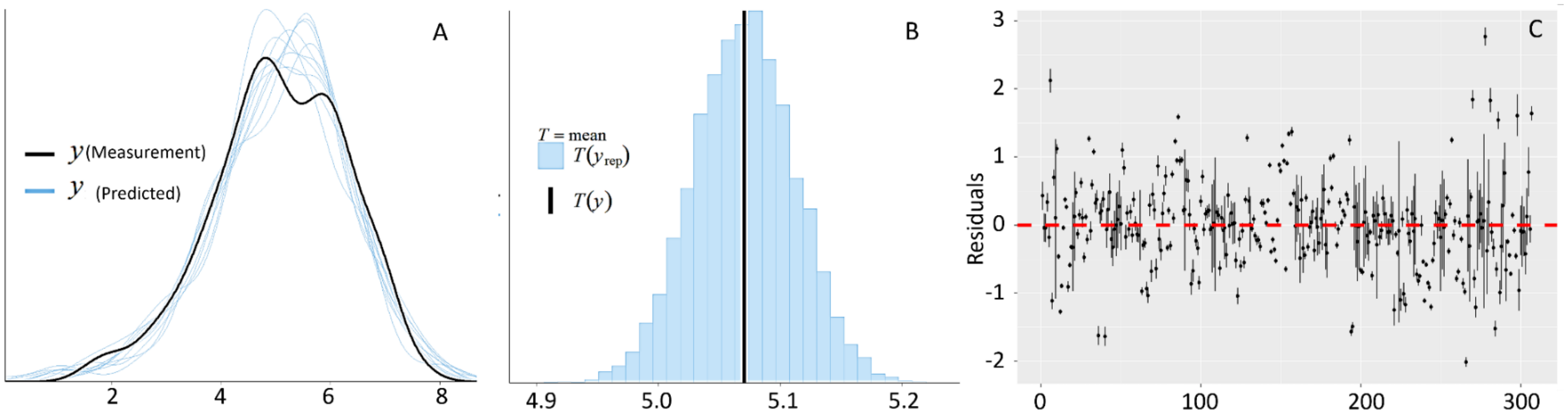

Figure S9. Posterior Predictive Check (PPC) for HAP-PM2.5 indoor concentration model

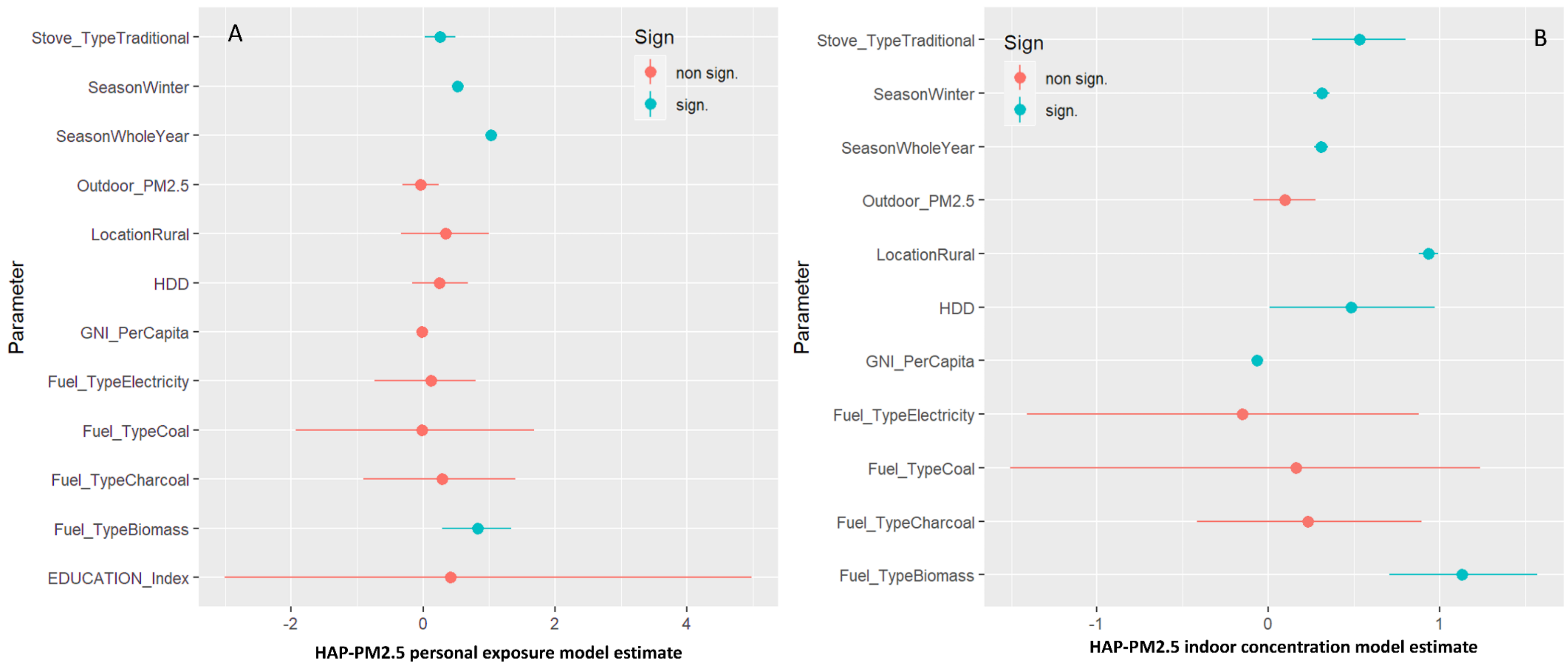

Figure S10. The fixed-effect posterior distribution statistics (coefficients from the model and the lower and upper 95% credible intervals). (A) HAP-PM2.5 personal exposure and (B) indoor concentration model coefficients. The significant level is given for p-value less than 0.05.

The degree of convergence of a random Markov Chain can be estimated using the Gelman-Rubin convergence statistic (Rhat). [34] Rhat evaluates Markov chain Monte Carlo (MCMC) convergence by analyzing the difference between multiple Markov chains. The convergence is assessed by comparing the estimated between-chains and within-chain variances for each model parameter. All values of Rhat are less than 1.01 indicate convergence to the underlying distribution. Values greater than 1.1 indicate inadequate convergence. Bulk-ESS is useful measure for sampling efficiency in the bulk of the distribution (related, for example, to efficiency of mean and median estimates), and is well defined even if the chains do not have finite mean or variance. Tail-ESS is useful measure for sampling efficiency in the tails of the distribution (related, for example, to efficiency of variance and tail quantile estimates). Both bulk-ESS and tail-ESS should be at least 100 (approximately) per Markov Chain to indicate that estimates of respective posterior quantiles are reliable.

Table S8. The fixed-effect posterior distribution statistics from the personal exposure model (coefficients, standard error, upper and lower 95% credible intervals and R-hat statistics as well as the Bulk Effective Sample Size (Bulk-ESS) and the Tail Effective Sample Size (Tail-ESS)

	Coefficient Estimate	Est. Error	Lower 95% CI	Upper 95% CI	Rhat	Bulk_ESS	Tail_ESS
Intercept	2.66	1.25	-0.32	4.96	1.005	643	602
LocationRural	0.34	0.33	-0.34	0.99	1.006	529	1009
Fuel_TypeBiomass	0.82	0.26	0.29	1.33	1.002	448	658
Fuel_TypeCharcoal	0.29	0.56	-0.90	1.39	1.004	525	748
Fuel_TypeCoal	-0.02	0.83	-1.93	1.68	1.002	1032	1269
Fuel_TypeElectricity	0.12	0.42	-0.74	0.79	1.003	462	383
Outdoor_$PM_{2.5}$	-0.04	0.13	-0.32	0.24	1.013	496	757
HDD	0.25	0.20	-0.16	0.67	1.010	355	668
Education_Index	0.41	1.90	-3.00	4.98	1.002	662	601
GNI_PerCapita	-0.01	0.03	-0.08	0.04	1.002	323	716
Stove_TypeTraditional	0.26	0.11	0.03	0.49	1.001	651	1012
SeasonWholeYear	1.03	0.00	1.01	1.05	1.002	1300	2574
SeasonWinter	0.52	0.00	0.50	0.54	1.002	1458	2711

Table S9. The fixed-effect posterior distribution statistics from the indoor concentration model (coefficient estimates, standard error, upper and lower 95% credible intervals and R-hat statistics as well as the Bulk Effective Sample Size (Bulk-ESS) and the Tail Effective Sample Size (Tail-ESS)

	Coefficient Estimate	Est.Error	Lower 95% CI	Upper 95% CI	Rhat	Bulk_ESS	Tail_ESS
Intercept	3.68	0.30	3.06	4.26	1.001	2726	4242
LocationRural	0.94	0.03	0.88	0.99	1.000	13242	6830
Fuel_TypeBiomass	1.13	0.22	0.71	1.57	1.000	2946	4457
Fuel_TypeCharcoal	0.23	0.33	-0.41	0.89	1.000	3417	4574
Fuel_TypeCoal	0.16	0.66	-1.50	1.23	1.001	4240	3728
Fuel_TypeElectricity	-0.15	0.57	-1.41	0.88	1.000	3793	3862
Stove_TypeTraditional	0.53	0.13	0.25	0.80	1.001	3690	5027
Outdoor_$PM_{2.5}$	0.09	0.09	-0.08	0.28	1.001	2375	3799
HDD	0.48	0.24	0.01	0.97	1.002	2081	3438
GNI_PerCapita	-0.06	0.02	-0.09	-0.03	1.000	3296	4651
SeasonWholeYear	0.31	0.02	0.27	0.35	1.000	10461	7037
SeasonWinter	0.31	0.02	0.27	0.36	0.999	10427	7551

Results of predicted annual average 24-hour HAP-$PM_{2.5}$ personal exposure µg/m^3, and attributable premature death rate for users of different fuel types, traditional stove technologies, and for urban and rural settings.

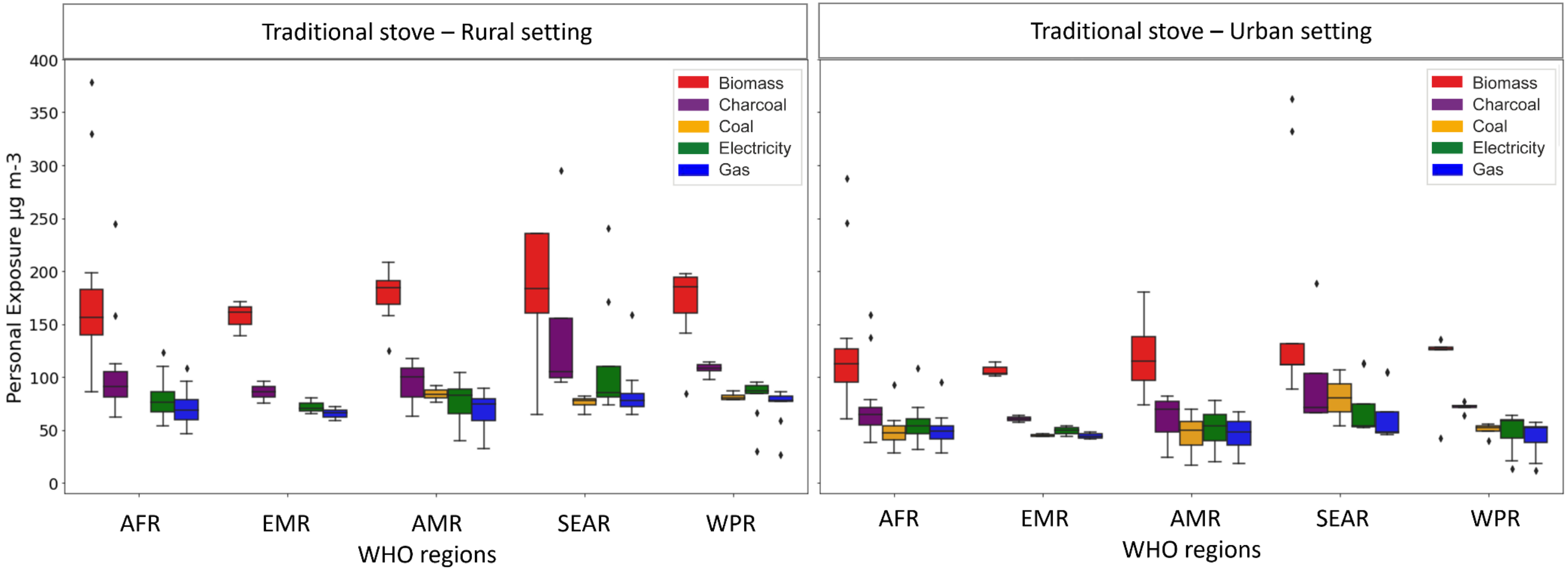

Figure S11. The predicted annual average 24-hour HAP-$PM_{2.5}$ personal exposure µg/m^3, for users of different fuel types, traditional stove technologies, and for urban and rural settings. The exposure results are aggregated for 5 WHO regions.

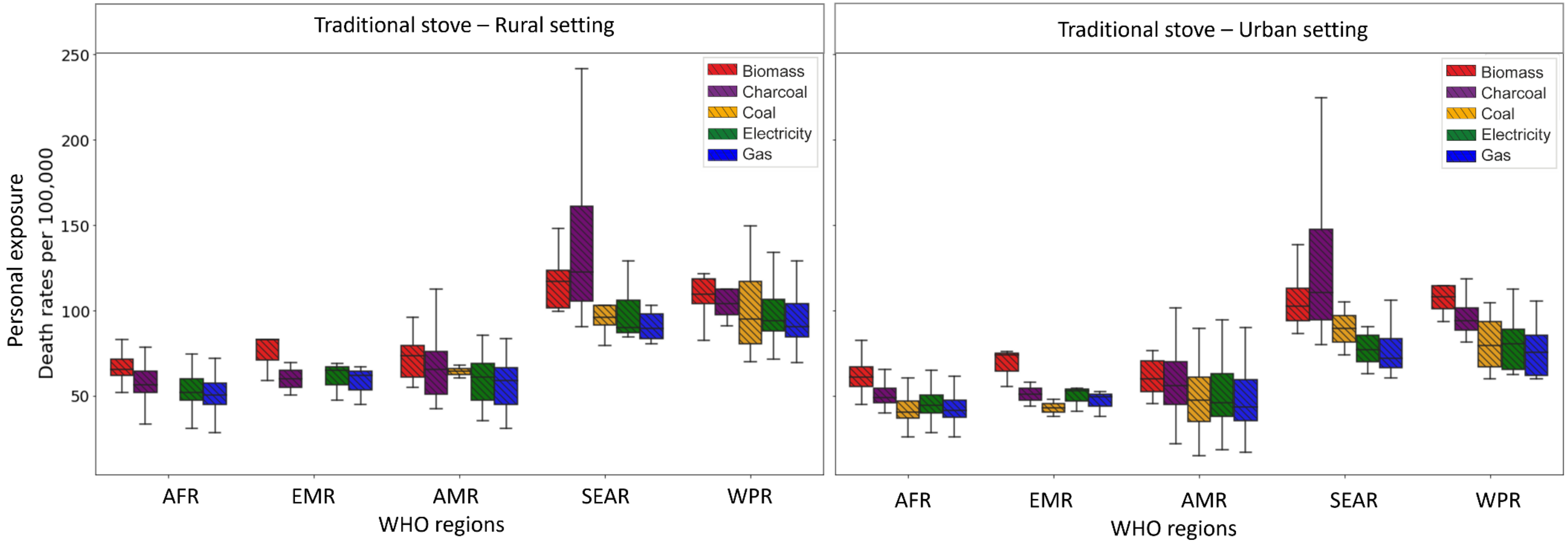

Figure S12. Attributable premature death rate (per 100, 000) population due to HAP-$PM_{2.5}$ personal exposure μg/m^3 for users of different fuel types, traditional stove technologies, and for urban and rural settings. The death rate results are aggregated for 5 WHO regions.

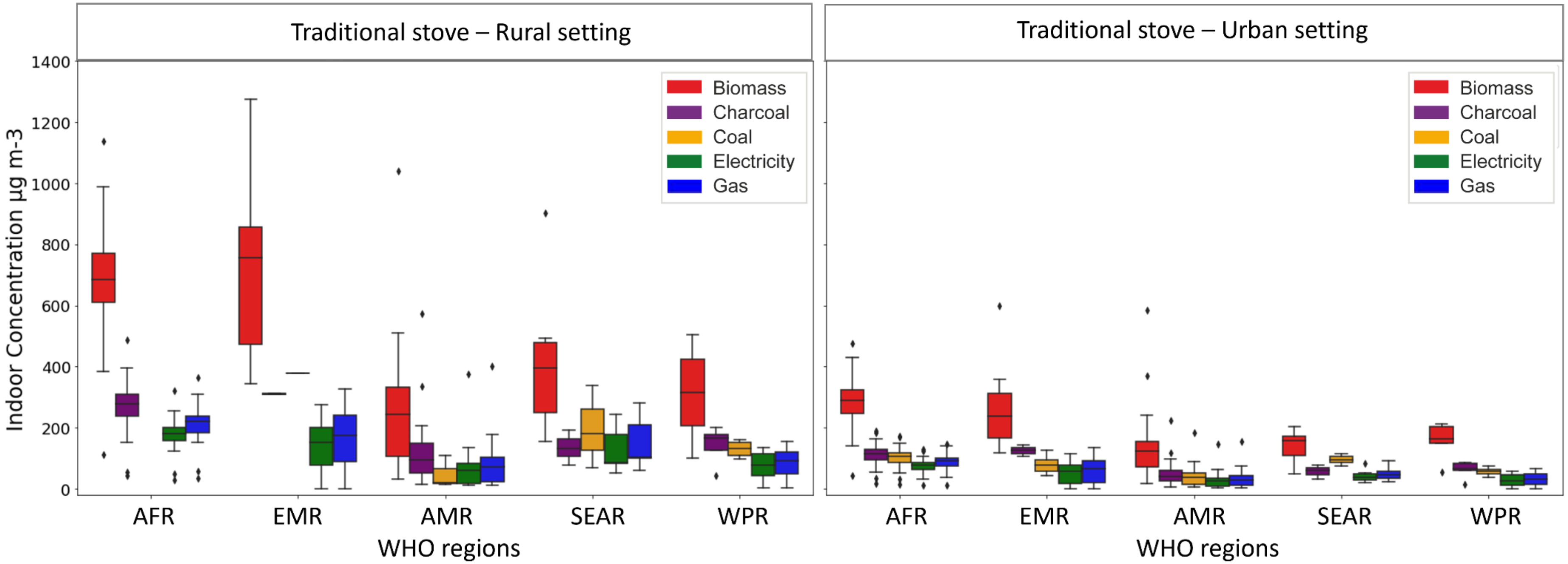

Figure S13. The predicted annual average 24-hour HAP-PM2.5 indoor concentration μg/m^3, for users of different fuel types, traditional stove technologies, and for urban and rural settings. The exposure results are aggregated for 5 WHO regions.

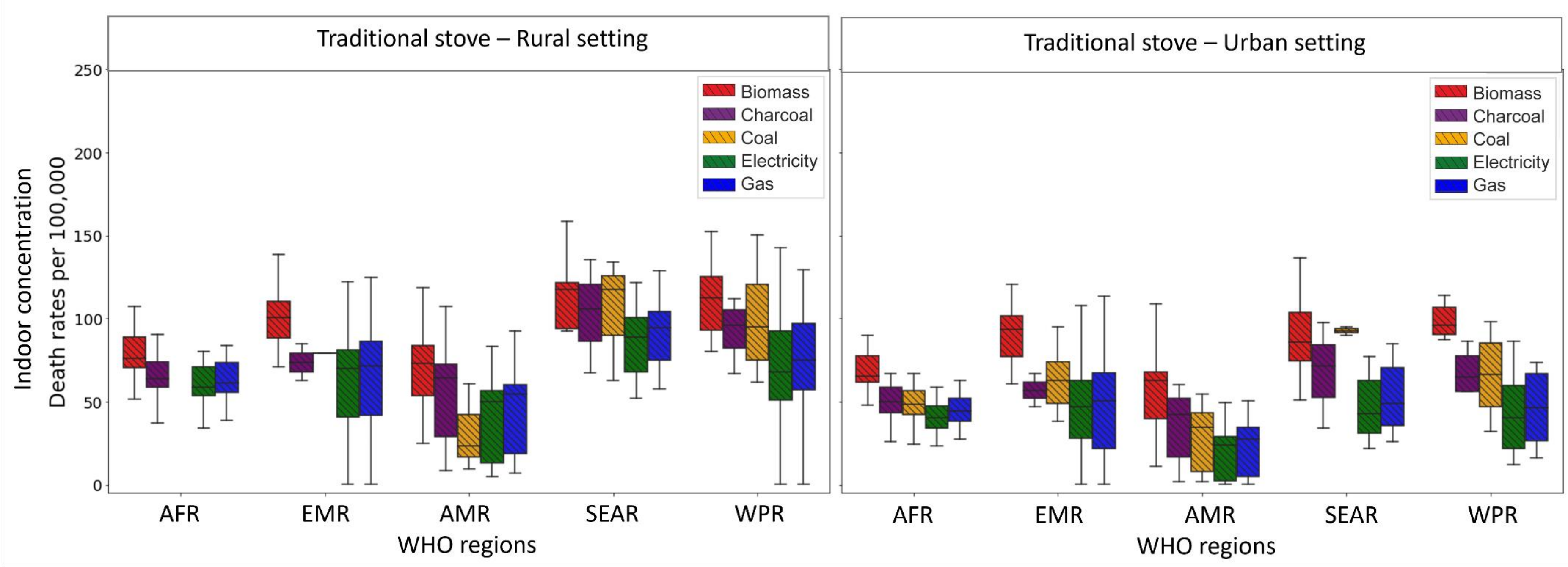

Figure S14. Attributable premature death rate (per 100, 000) population due to HAP-$PM_{2.5}$ indoor concentration $\mu g/m^3$ for users of different fuel types, traditional stove technologies, and for urban and rural settings. The death rate results are aggregated for 5 WHO regions. We multiplied the $PM_{2.5}$ indoor concentration (figure S13) by a factor of 0.6 to approximate a time-averaged indoor exposure when estimating attributable premature death rate due to indoor concentration, assuming that, on average, 60% of the time is spent indoors at home. % of time spent inside houses depends on several factors: (i) people living in the rural and urban locations, (ii) people living in developing countries and developed countries, (iii) gender, including women, men and children (going to school or going to work). As we have limited information about the above factors for each country [35], our estimated 60% on average is based on an assumption of a typical person who spends about 2/3 of his/her time at home.

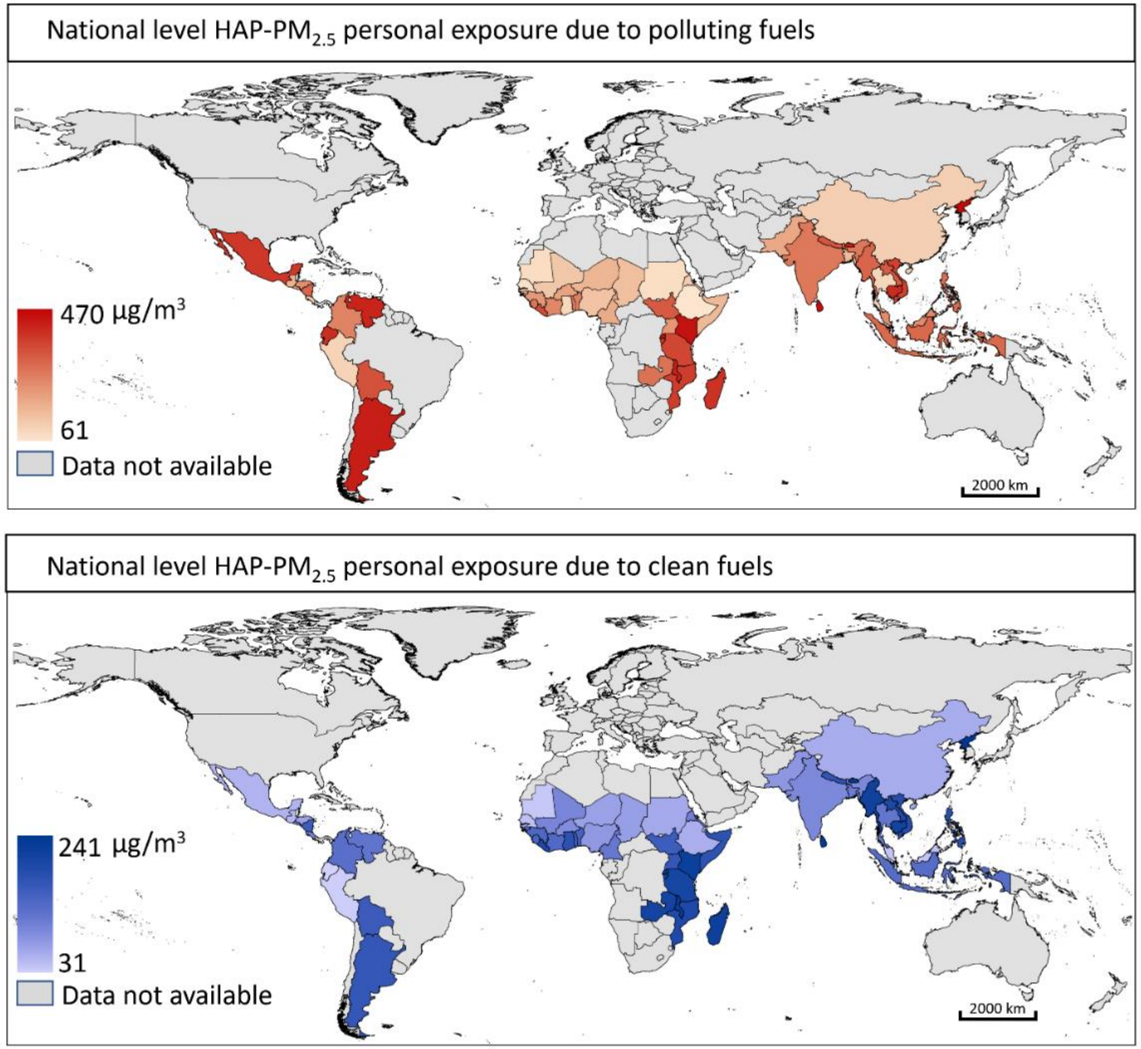

Figure S15. The national-level annual weighted average 24-hour HAP-$PM_{2.5}$ personal exposure due to polluting and clean fuels for 62 countries

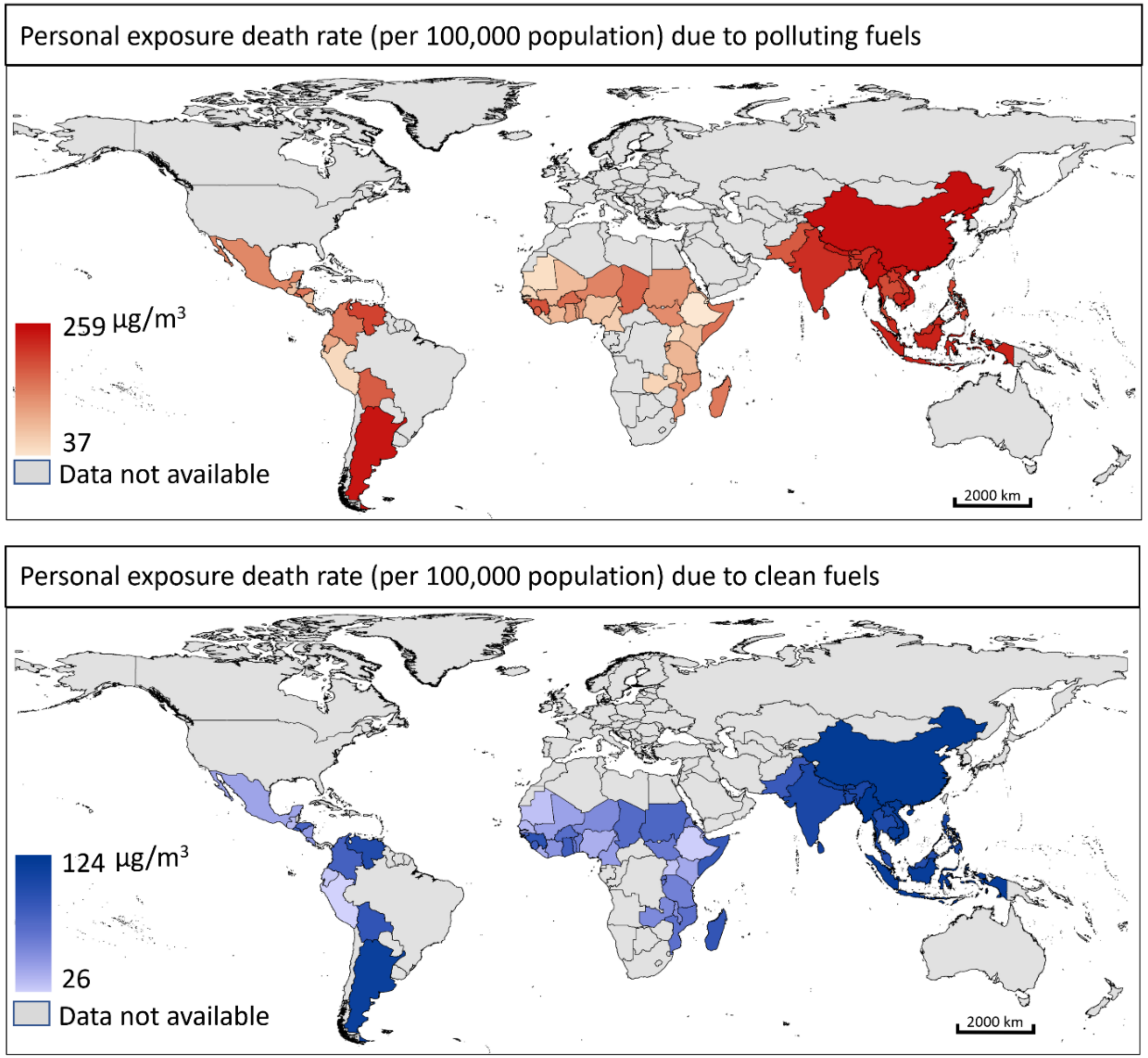

Figure S16. The national level personal exposure attributable premature death rate (per 100,000 population) due to polluting and clean fuels for 62 countries

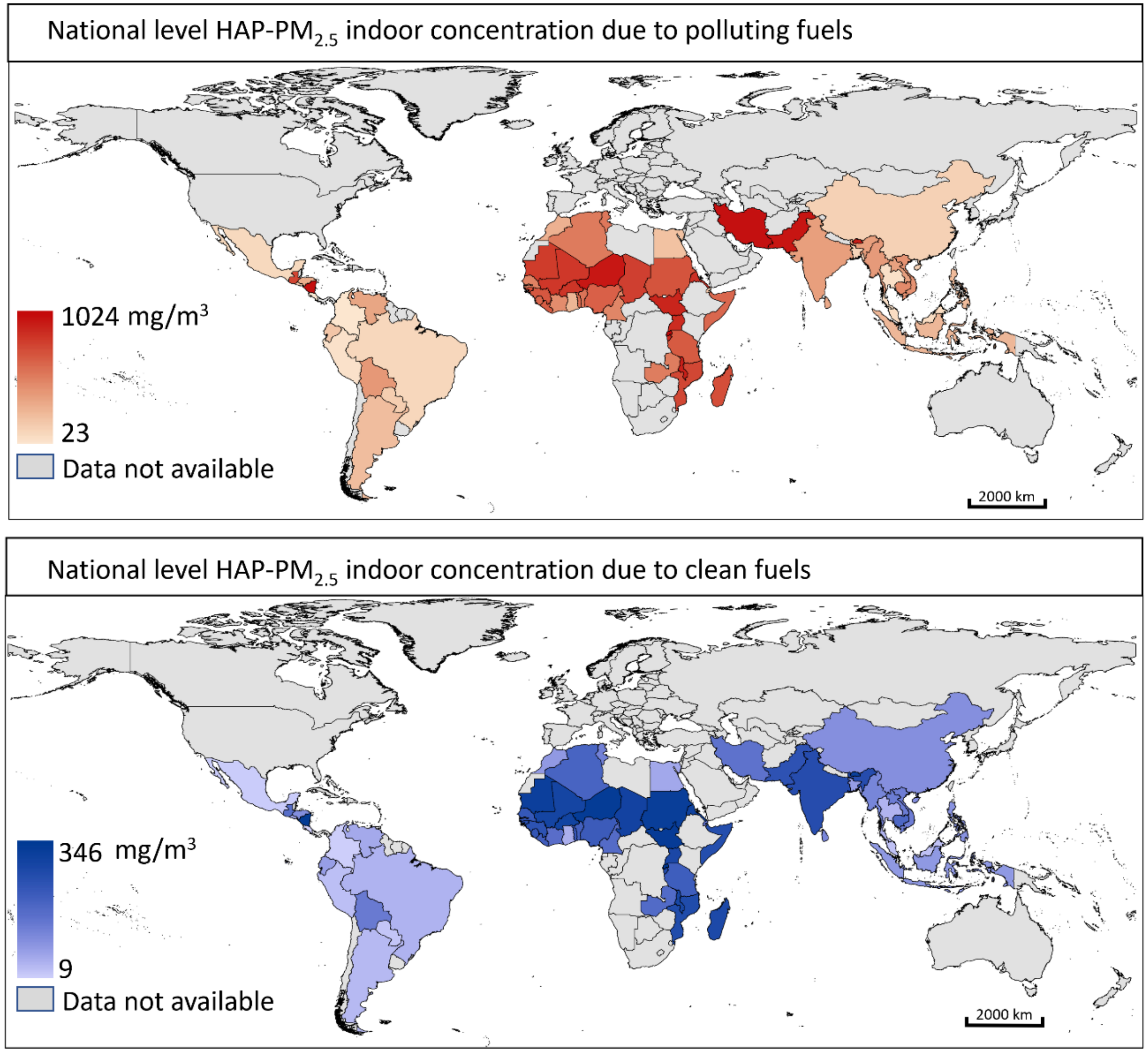

Figure S17. The national level annual weighted average 24-hour HAP-$PM_{2.5}$ indoor concentration due to polluting and clean fuels for 69 countries

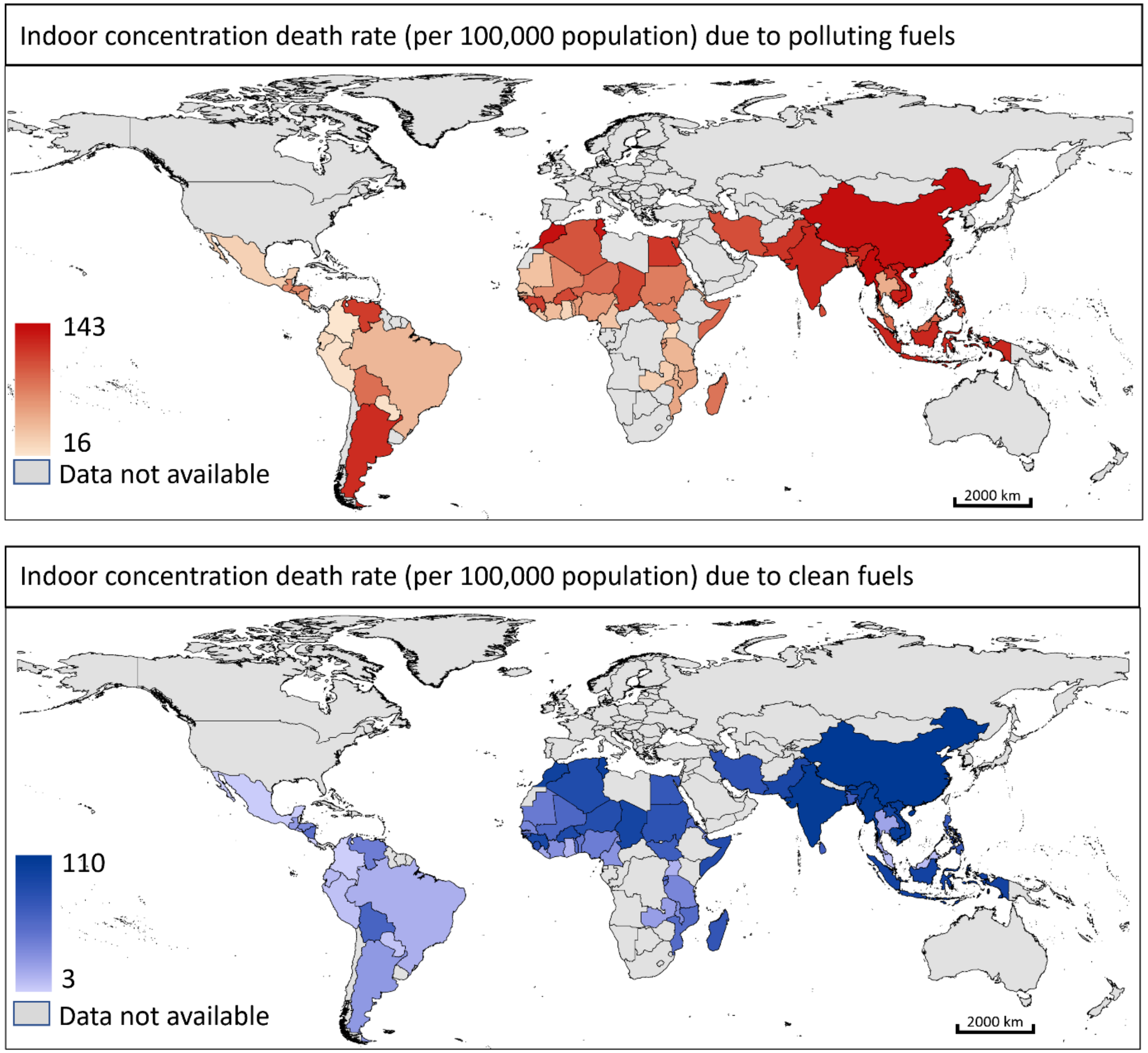

Figure S18. The national level indoor concentration attributable premature death rate (per 100,000 population) due to polluting and clean fuels for 69 countries

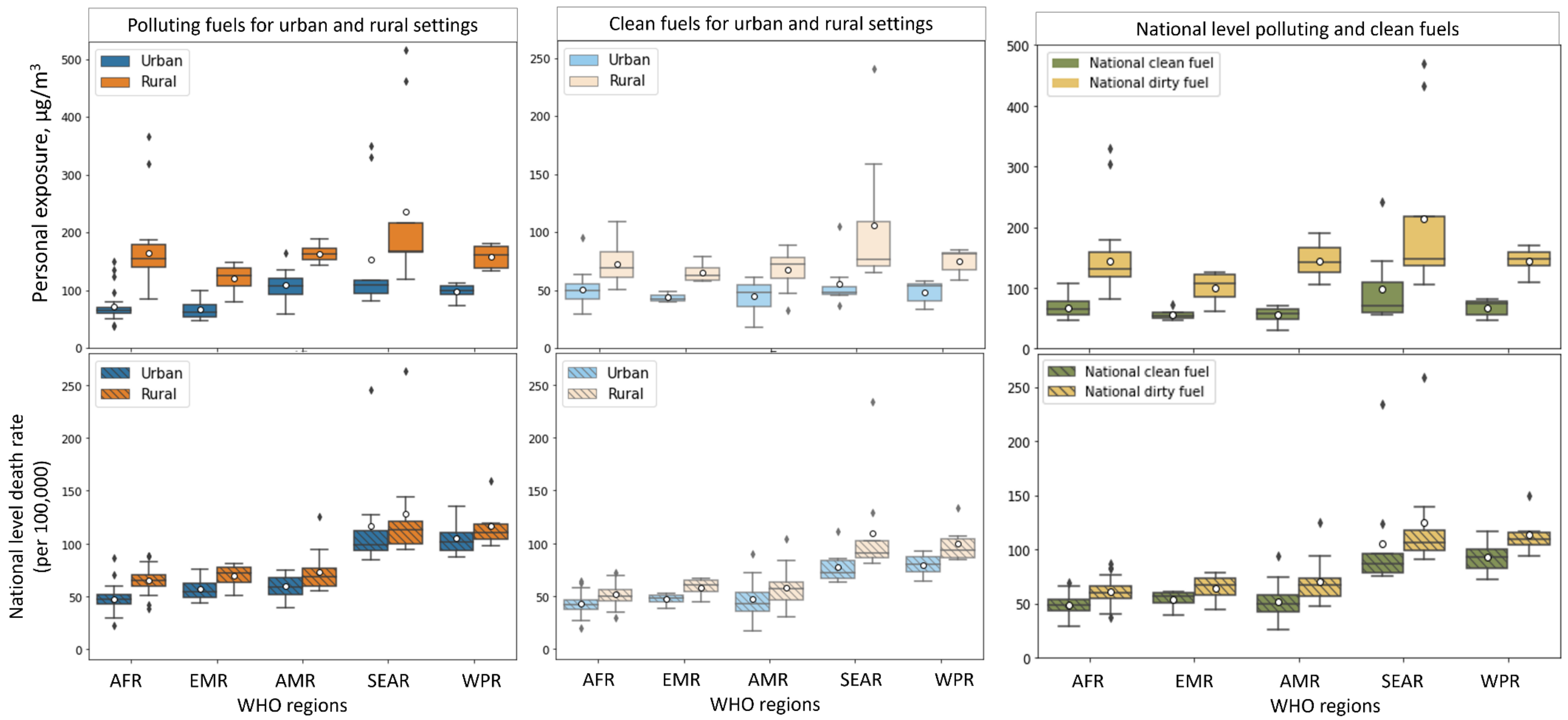

Figure S19. The urban, rural, and national level annual weighted average, median, quartiles, and outliers for 24-hour HAP-PM2.5 personal exposure and related attributable premature death rate (per 100,000 population) of polluting solid fuels and clean fuels for 5 WHO regions. AFR (African Region), AMR (Region of the Americas), SEAR (South-East Asian Region), WPR (Western Pacific Region), Eastern Mediterranean Region (EMR).

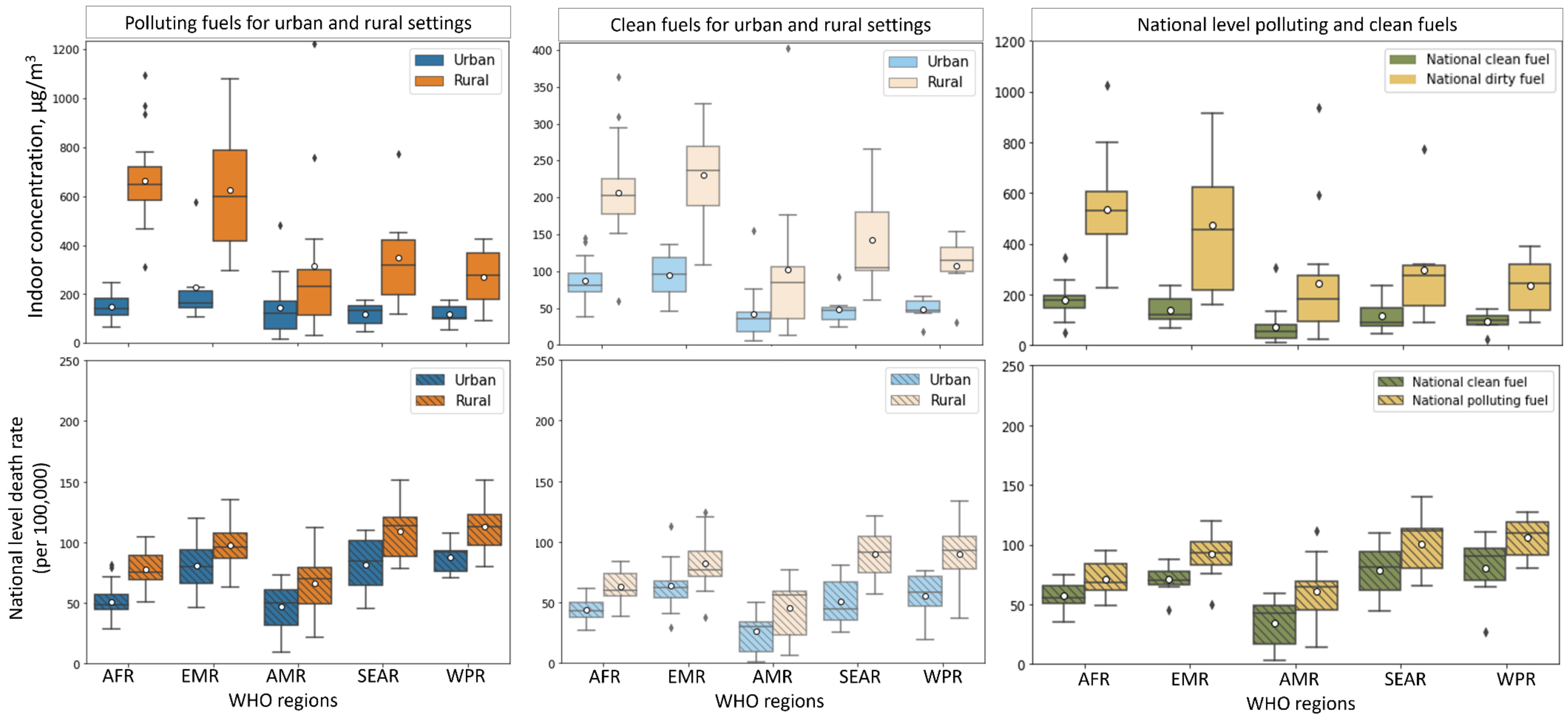

Figure S20. The urban, rural, and national level annual weighted average, median, quartiles, and outliers for 24-hour HAP-$PM_{2.5}$ indoor concentration and related attributable premature death rate (per 100,000 population) of polluting solid fuels and clean fuels for 5 WHO regions. AFR (African Region), AMR (Region of the Americas), SEAR (South-East Asian Region), WPR (Western Pacific Region), Eastern Mediterranean Region (EMR).

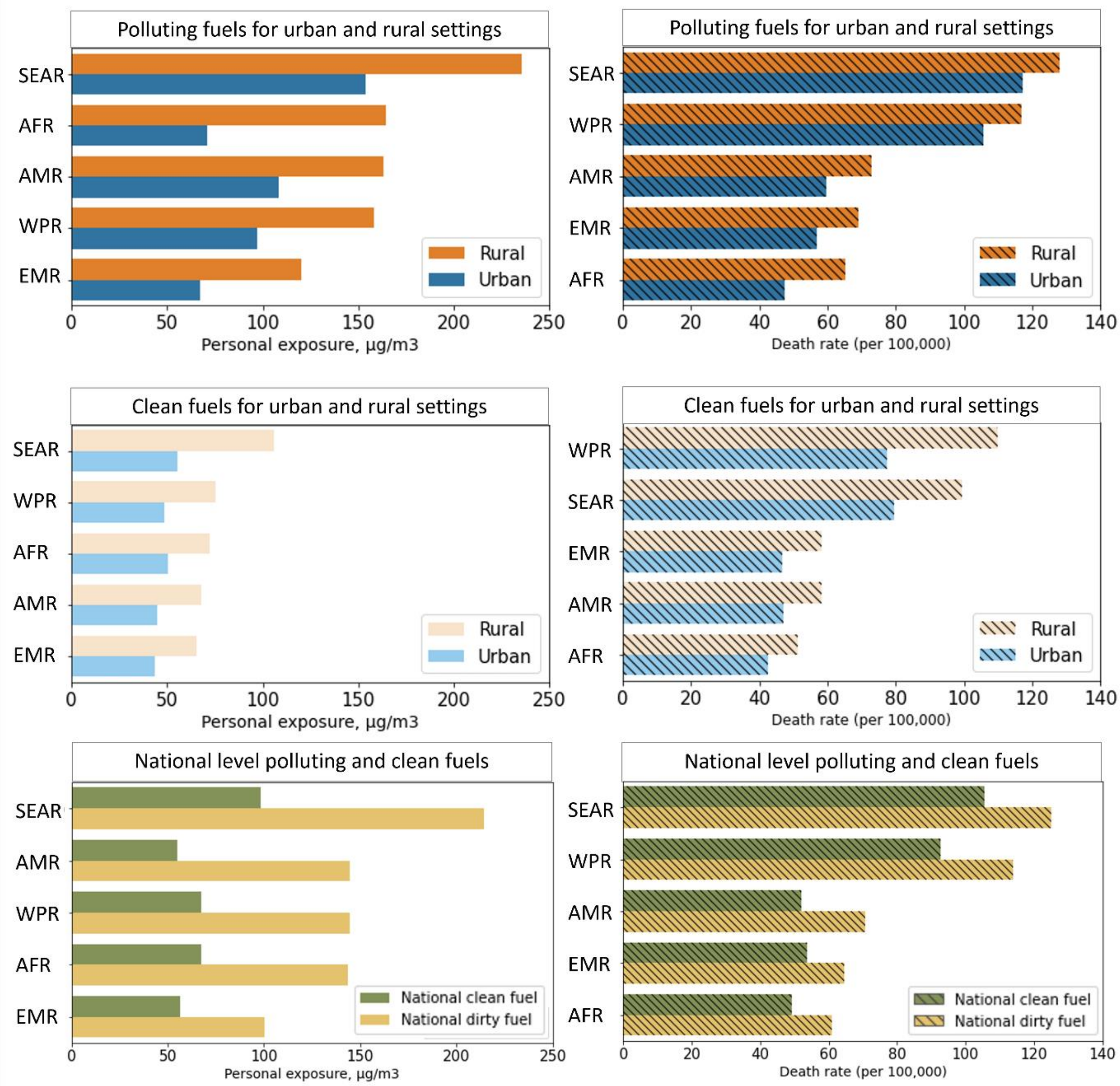

Figure S21. The urban, rural, and national level annual weighted average 24-hour HAP-PM2.5 personal exposure and related attributable premature death rate (per 100,000 population) of polluting solid fuels and clean fuels for 5 WHO regions. AFR (African Region), AMR (Region of the Americas), SEAR (South-East Asian Region), WPR (Western Pacific Region), Eastern Mediterranean Region (EMR).

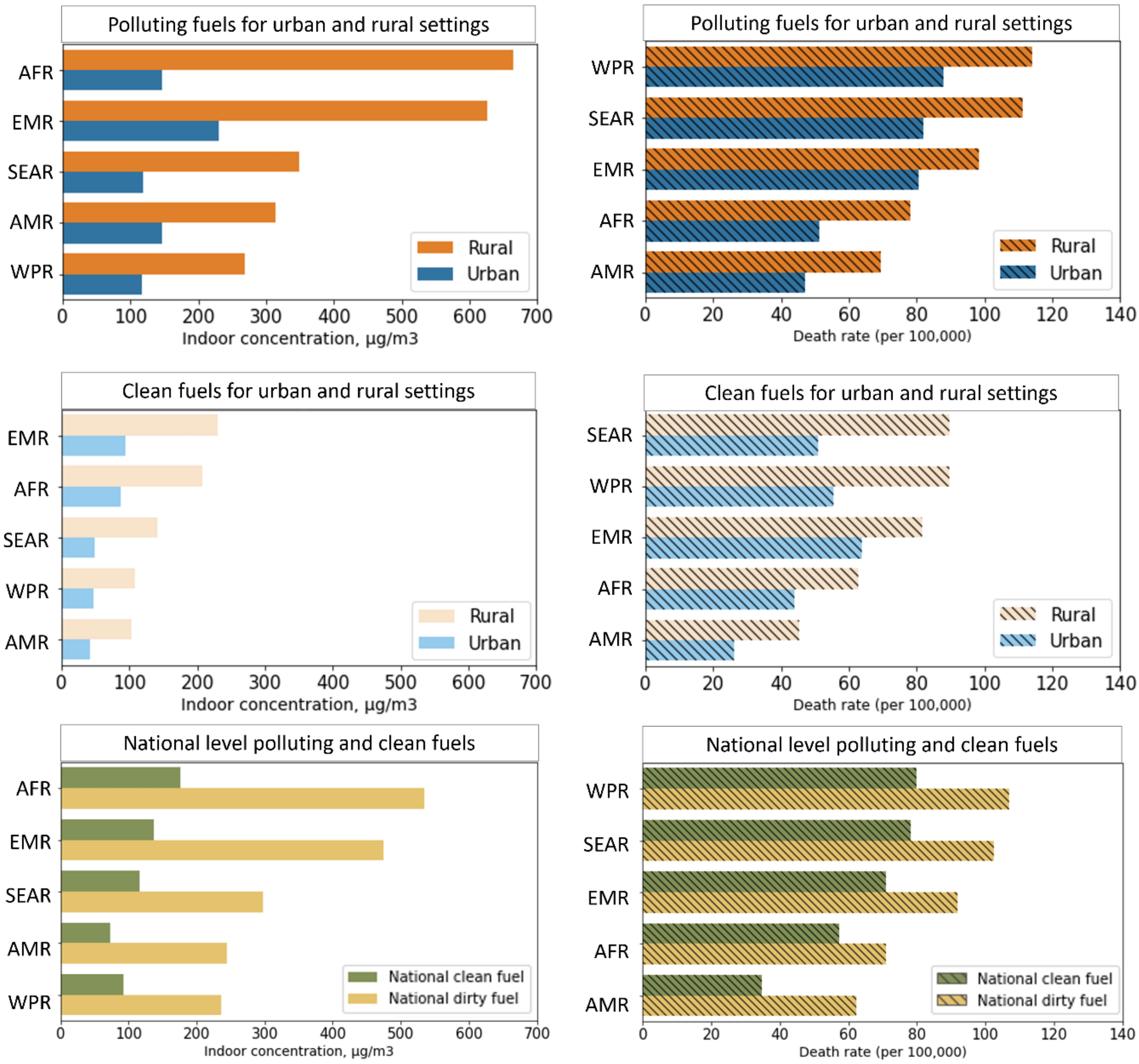

Figure S22. The urban, rural, and national level annual weighted average 24-hour HAP-PM2.5 indoor concentration and related attributable premature death rate (per 100,000 population) of polluting solid fuels and clean fuels for 5 WHO regions. AFR (African Region), AMR (Region of the Americas), SEAR (South-East Asian Region), WPR (Western Pacific Region), Eastern Mediterranean Region (EMR).

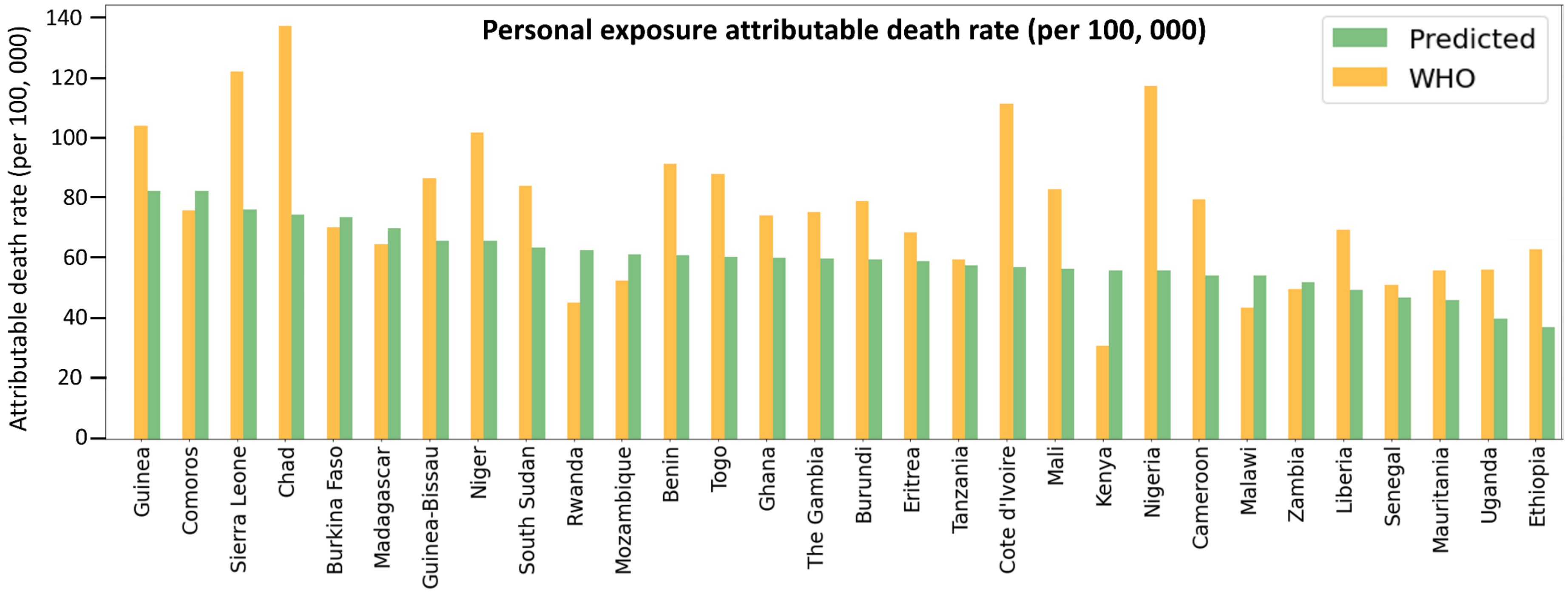

Figure S23. The annual personal exposure attributable death rate (per 100,000) for individuals using solid fuels (biomass, charcoal, coal) at national level compared to those from WHO [36] for countries from African Region (estimated 30 countries out of 62 countries in African Region) [37].

Reference

1. Shupler M, Hystad P, Birch A, et al. Household and personal air pollution exposure measurements from 120 communities in eight countries: results from the PURE-AIR study. *Lancet Planetary Health* 2020; 4: e451–62.
2. Shupler M, Hystad P, Birch A, et al. Multinational prediction of household and personal exposure to fine particulate matter ($PM_{2.5}$) in the PURE cohort study. *Environment International 2022;* 159: 107021.
3. Shupler M., Godwin W, Frostad J, et al. Global estimation of exposure to fine particulate matter (PM2.5) from household air pollution. Environment International 2018; 120: 354-363.
4. Li Q, Jiang J, Wang S, et al. Impacts of household coal and biomass combustion on indoor and ambient air quality in China: current status and implication. *Sci. Total Environ. 2017;* 576: 347-361, 10.1016/j.scitotenv.2016.10.080
5. WHO. Ambient (outdoor) air pollution. WHO Fact Sheets. Geneva: World Health Organization, 2021. Available from: https://www.who.int/news-room/fact-sheets/detail/ambient-(outdoor)-air-quality-and-health. (accessed December 10, 2021)
6. Stoner O, Lewis J, Martinez IL. et al. 2021. Household cooking fuel estimate at the global and county level for 1990 to 2030. *Nature communications* 2021; 12: 5793.
7. Balakrishnan K, Ghosh S, Ganguli B, et al. State and national household concentrations of PM2.5 from solid cookfuel use: results from measurements and modeling in India for estimation of the global burden of disease. *Environ Health* 2013; 12:77. Doi: https://doi.org/10.1186/1476-069X-12-77.
8. WHO. Global database of household air pollution measurements. Geneva: World Health Organization, 2018. https://www.who.int/data/gho/data/themes/air-pollution/hap-measurement-db. (accessed June10, 2021)
9. Pope CA, Burnett RT, Krewski D, et al. Cardiovascular mortality and exposure to airborne fine particulate matter and cigarette smoke: shape of the exposure-response relationship. *Circulation* 2009; 120: 941-948, 10.1161/CIRCULATIONAHA.109.857888
10. Pope CA, Burnett RT, Turner MC, et al. Lung cancer and cardiovascular disease mortality associated with ambient air pollution and cigarette smoke: shape of the exposure–response relationships. *Environ. Health Perspect*. 2011; 119:1616-1621, 10.1289/ehp.1103639
11. Smith KR, Peel JL. Mind the gap. *Environ. Health Perspect*. 2010; 118: 1643-1645, https://doi.org/10.1289/ehp.1002517
12. Lim SS, Vos T, Flaxman AD, et al. A comparative risk assessment of burden of disease and injury attributable to 67 risk factors and risk factor clusters in 21 regions, 1990–2010: a systematic analysis for the Global Burden of Disease Study 2010. The Lancet 2013; 380: 2224–2260. Doi: https://doi.org/10.1016/S0140-6736(12)61766-8
13. Murray CJ, Ezzati M, Flaxman AD, et al. GBD 2010: design, definitions, and metrics. *The Lancet* 2012; 380: 2063-2066, 10.1016/S0140-6736(12)61899-6
14. Smith K. National Burden of Disease in India from Indoor Air Pollution. *Proceedings of the National Academy of Sciences of the United States of America* 2000; 97: 13286-13293. Retrieved May 18, 2021, from http://www.jstor.org/stable/123694
15. Forouzanfar MH, Alexander L, Anderson HR, et al. Global, regional, and national comparative risk assessment of 79 behavioural, environmental and occupational, and metabolic risks or clusters of risks in 188 countries, 1990–2013: a systematic analysis for the Global Burden of Disease Study 2013. The Lancet 2015; 386:2287–2323. Doi: https://doi.org/10.1016/S0140-6736(15)00128-2
16. Gakidou E, Abate KH, Abbafati C, et al. Global, regional, and national comparative risk assessment of 84 behavioural, environmental and occupational, and metabolic risks or clusters of risks, 1990–2016: a systematic analysis for the Global Burden of Disease Study 2016. *The Lancet* 2017; 390:1345–1422. Doi: https://doi.org/10.1016/S0140-6736(17)32366-8
17. Shupler M, Balakrishnan K, Ghosh S, et al. Global household air pollution database: Kitchen concentrations and personal exposures of particulate matter and carbon monoxide. *Data Brief* 2018; 27: 21:1292-1295. doi: 10.1016/j.dib.2018.10.120. PMID: 30456246; PMCID: PMC6231029.
18. Meta (a Toronto-based artificial intelligence scientific literature search engine). Available from chanzuckerberg.com. (accessed June10, 2021)

19. NASA Earth Exchange Global Daily Downscaled Projections (NEX-GDDP). 2000. Available from: https://www.nccs.nasa.gov/services/data-collections/land-based-products/nex-gddp-cmip6
20. OpenAQ. Available from www.openaq.org
21. International Energy Agency (IEA). 2021. Financing clean energy transitions in emerging and developing economies. Paris: International Energy Agency, 2021. https://www.iea.org/reports/financing-clean-energy-transitions-in-emerging-and-developing-economies (accessed June 2022).
22. Klimont Z, Cofala J, Bertok I et al. (2002) Modelling Particulate Emissions in Europe A Framework to Estimate Reduction Potential and Control Costs. Interim Report, IR-02-076. Luxemburg: International Institute for Applied Systems Analysis, 2002
23. Klimont, Z., Kupiainen, K., Heyes, C., Purohit, P., Cofala, J., Rafaj, P., Borken-Kleefeld, J., and Schöpp, W.: Global anthropogenic emissions of particulate matter including black carbon, Atmos. Chem. Phys., 17, 8681–8723, https://doi.org/10.5194/acp-17-8681-2017, 2017
24. Amann M et al. 2020, Reducing global air pollution: the scope for further policy interventions. Phil. Trans. R. Soc. A 378: 20190331, http://dx.doi.org/10.1098/rsta.2019.0331
25. Romanello M, McGushin A, Di Napoli C, et al. The 2021 report of the Lancet Countdown on health and climate change: code red for a healthy future. The Lancet 2021; 398(10311): 1619-62.
26. Global Human Settlement Layer (GHSL). 2015. Available from: https://ghsl.jrc.ec.europa.eu/datasets.php.
27. United Nations Development Programme (UNDP), 2020. Human Developments Reports. Available from: https://hdr.undp.org/data-center/documentation-and-downloads
28. World Health Organization (WHO). 2022. Household Air Pollution and Health. WHO Fact Sheets, 2022. Available from: https://www.who.int/news-room/fact-sheets/detail/household-air-pollution-and-health
29. Southerland V, Braver M, Mohegh A et al., 2022. Global urban temporal trends in fine particulate matter (PM2·5) and attributable health burdens: estimates from global datasets. The Lancet Planetary Health 6, E139-E146.
30. Billie L. Taylor. 1981. Population-weighted heating degree-days for Canada. Atmosphere-Ocean, 19:3, 261-268, DOI: 10.1080/07055900.1981.9649113
31. McNeish D. On using Bayesian methods to address small sample problems. *Structural Equation Modelling* 2016; 23: 750–773. doi: 10.1080/10705511.2016. 1186549.
32. LeMoine NP. Moving Beyond Noninformative Priors: Why and How to Choose Weakly Informative Priors in Bayesian Analyses Weakly Informative Priors in Bayesian Analyses. *Oikos* 2019; 128: 912–928, doi: 10.1111/oi k .05985.
33. Vehtari A, Gelman A, Gabry, J. Practical Bayesian model evaluation using leave-one-out cross-validation and WAIC. *Statistics and Computing* 2017; 27: 1413–1432. https://doi.org/10.1007/s11222-016-9696-4.
34. Vehtari A, Gelman A, Simpson D, et al. Rank-normalization, folding, and localization: An improved *R-hat* for assessing convergence of MCMC. *arXiv preprint* 2019: *arXiv:1903.08008*.
35. Klepeis N, Nelson W, Ott W., et al. 2001. The National Human Activity Pattern Survey (NHAPS): a resource for assessing exposure to environmental pollutants. J Expo Sci Environ Epidemiol 11, 231–252 (2001). https://doi.org/10.1038/sj.jea.7500165
36. WHO. Household air pollution attributable death rate (per 100 000 population). Available from: https://www.who.int/data/gho/data/indicators/indicator-details/GHO/household-air-pollution-attributable-death-rate-(per-100-000-population). (accessed June10, 2022)
37. Romanello M, Di Napoli C, Drummond P, et al. The 2022 report of the Lancet Countdown on health and climate change: health at the mercy of fossil fuels. The Lancet 2022; DOI:https://doi.org/10.1016/S0140-6736(22)01540-9.
